# Exploiting the Hessian for a Better Convergence of the SCF-RDMFT Procedure

**DOI:** 10.1021/acs.jctc.4c00118

**Published:** 2024-04-26

**Authors:** Nicolas G. Cartier, Klaas J. H. Giesbertz

**Affiliations:** Department of Chemistry & Pharmaceutical Sciences and Amsterdam Institute of Molecular and Life Sciences (AIMMS), Faculty of Science, Vrije Universiteit, De Boelelaan 1083, 1081 HV Amsterdam, The Netherlands

## Abstract

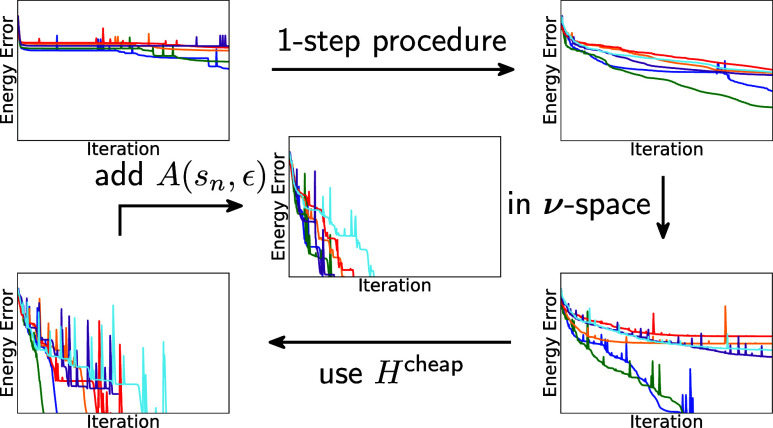

One-body reduced density matrix functional theory provides an alternative to density functional theory, which is able to treat static correlation while keeping a relatively low computation scaling. Its disadvantageous cost comes mainly from a slow convergence of the self-consistent energy optimization. To improve on that problem, we propose in this work the use of the Hessian of the energy, including the coupling term. We show that using the exact Hessian is very effective at reducing the number of iterations. However, since the exact Hessian is too expensive to use in practice, we propose an approximation based on an inexpensive exact part and BFGS updates.

## 1 Introduction

Density functional theory (DFT) has been widely used to compute the ground-state energy of electronic systems over the past few decades because of its advantageous trade-off between cost and accuracy.^1−7^ However, it can fail already qualitatively, especially for systems with large static correlations.^8−15^ A promising choice to improve the accuracy in these cases, while keeping a reasonable computation scaling, is to use one-body reduced density matrix functional theory (RDMFT),^16−18^ which naturally describes static correlations.^19−25^ The large cost of the self-consistent field (SCF) minimization of the ground-state energy in RDMFT (SCF-RDMFT) remains, nevertheless, an obstacle to its acceptance by the community.^17,26,27^ The large cost is mostly incurred by the exceptionally large number of iterations. The primary focus of this work is then to reduce the number of SCF iterations to bring RDMFT closer as a practical alternative for cases in which DFT fails.

DFT relies on the Hohenberg–Kohn theorem,^28^ which proves that the total energy can be written as a functional of the density for *v*-representable densities. A decade later, Gilbert used the same approach to show that the total energy can also be expressed as a functional of the one-body reduced density matrix (1-RDM) γ, for *v*-representable 1-RDMs.^29^ The advantage is that more general perturbations can be considered than only local potentials, e.g., magnetic fields, and that the kinetic energy is a simple linear functional of the 1-RDM. The total energy functional then reads
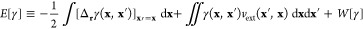
1where **x**_*i*_ = **r**_*i*_σ_*i*_ denotes a combined space-spin coordinate, Δ_**r**_ the Laplacian with respect to **r**, *v*_ext_ the (non)local external potential, and *W* the interaction energy functional, which Gilbert defined as
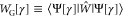
2with Ψ, an *N*_*e*_-particle wave function, and *Ŵ*, an electron–electron interaction operator, which is the Coulomb interaction in practice. Note the similarity with the Hohenberg–Kohn functional in DFT and also the striking difference that the kinetic energy is not part of the universal functional, so “only” the interaction energy, for which we do not have an exact explicit expression in terms of the 1-RDM, thus needs to be approximated.

However, Gilbert’s approach suffers from the same problem as the Hohenberg–Kohn theorem: in order to minimize the functional, one needs to remain in the domain on which the functional is defined (domain of *v*-representable 1-RDMs^30,31^), which is unknown in general. This problem was partially solved by Levy,^32^ who extended the functional to the domain of pure state *N*-representable 1-RDMs, i.e., 1-RDMs, which can be generated by an *N*_*e*_-particle wave function via
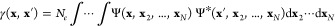
3

The universal functional is then defined via the constrained search construction as
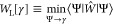
4

Contrary to DFT, a complete characterization of the domain of pure state *N*-representable 1-RDMs in terms of explicit conditions on the 1-RDM is nonetheless difficult to obtain.^33−36^ Valone therefore generalized Levy-constrained search to the domain of ensemble *N*-representable 1-RDMs^37^
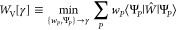
5

The prescription {*w*_*P*_, Ψ_*P*_} → γ means that the ensemble composed of states {Ψ_*P*_} with respective weights {*w*_*P*_} generates the 1-RDM via^38^
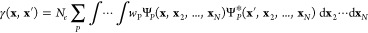
6where *w*_*P*_ ≥ 0 and *∑*_*P*_*w*_*P*_ = 1. The functional *W*_V_[γ] also has the nice property of being convex.^39,40^

To ensure that a 1-RDM is ensemble *N*-representable, it is sufficient to impose that Tr{γ} = *N*_*e*_, γ and *I* – γ are positive semidefinite (*I*, denoting the identity matrix).^38^ In order to impose these constraints, most SCF procedures in RDMFT work directly within the basis which diagonalizes the 1-RDM^26,41−43^
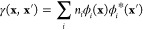
7where the eigenvalues *n*_*i*_ are called the (natural) occupation numbers (ONs) and the eigenfunctions ϕ_*i*_(**x**), the natural orbitals (NOs).^44^ Moreover, most approximate RDMFT functionals are defined in this basis.^21,24,45−49^

There are several ways to enforce the inequality constraints on the ONs, arising from ensemble *N*-representability, and in particular, parameterization by some suitable function of a variable *x* seems to be a popular choice. One of the earliest parameterizations is the cos(*x*)^2^ function,^29,50−52^ though the Fermi–Dirac function 1/(*e*^–β*x*^ + 1)^27,43^ and the error function erf(*x*)^53^ are perhaps more attractive choices. Other authors opt for more direct optimization with the Powell^41^ or Lagrange multiplier method, in particular also for the constraint on the trace.^19,26^ One can then optimize the ONs using standard methods like conjugate-gradient^51,52^ or (quasi-)Newton methods.^27,52−54,54,55^

One also has to impose the orthonormality of the NOs. The most popular way, to do so, is to optimize the NOs using an iterative diagonalization of a generalized Fock matrix.^19,26,27,42,43^ Another common way is to write the NOs as a unitary transformation *U* of an orthonormal basis. The most standard form is *U* = exp(*X*), taking *X* as a real antisymmetric matrix.^17,56,57^ Some authors also choose to preserve orthonormality only approximately to calculate an optimization step and reorthonormalize the NOs afterward^41,50,52,58^ or brute forcefully apply a Lagrange multiplier method.^59^

Most approaches described here are based on a two-step scheme, in which the ONs and NOs are each optimized in turn. The main disadvantage is that no information on the coupling between the NOs and ONs is used, so typically a large number of macroiterations (i.e., one pass over an ON optimization followed by an NO optimization) are needed to obtain consistency between the NOs and ONs. A notable exception investigating a simultaneous optimization of NOs and ONs with the preconditioned conjugate-gradient method was however published during the review process of the present work,^60^ though that work still does not include the coupling between ON–NO within a step.

A second-order single-step method which optimizes the NOs and ONs simultaneously therefore seems to be a sensible direction. The coupling between the NOs and ONs is naturally included via the off-diagonal ON–NO blocks of the Hessian in a (quasi-)Newton scheme. An additional reason to use the Hessian is that the unitary parameterization of the NOs *U*(*X*) yields a nonconvex energy functional in terms of the parameters *X* (even if the functional is convex in γ, e.g., the exact RDMFT functional). The energy landscape becomes much more bumpy in *X* parameter space, and the Hessian provides very useful information to guide the optimization algorithm through curved valleys. Since it is not straightforward to include second-order information in the iterative diagonalization approach, we focus in this work on the orthogonalization of the NOs via a parameterization by a unitary matrix.

The aim of this work is to reduce the total number of iterations required for the SCF procedure to converge to arbitrary precision. To achieve this goal, we proceed as follows. After giving some details on the implementation in Section 2 and deriving the expression of the exact Hessian in Section 3, we look at the waste of optimization steps that can arise when optimizing the ONs and NOs separately, in Section 4. Then we try to extract as much information as possible from the exact Hessian in Section 5. Third, we introduce an intermediate set of variables and combine it with our second point to obtain an approximation of the Hessian in Section 6. We finally conclude in Section 7.

## 2 Numerical Details

The purpose of this research is to improve the SCF convergence, we want to avoid additional difficulties coming from the functionals themselves as much as possible. To do so, we have decided to test our methods exclusively on the Müller functional, also known as the Buijse–Baerends (BB) functional,^61,62^ known for its convexity with respect to the 1-RDM.^63^ For the Müller functional
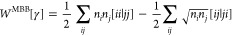
8with
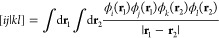
9where the orbitals {ϕ_*i*_} have been taken real as we work in a nonrelativistic setting and restrictive to the spin-summed case, where 0 ≤ *n*_*i*_ ≤ 2, from now on.

As mentioned in the Introduction, though the Müller functional is convex with respect to the 1-RDM, the unitary parameterization *U*(*X*) yields a functional which is not convex with respect to *X*. Thus, the Hessian is generally indefinite even for this simple functional. It is therefore preferable to turn to methods able to handle indefinite Hessians, such as trust-region methods.^64,65^ We then work with a trust-region (quasi-)Newton algorithm. As the cost of the method will be dominated by the amount of evaluations of the functional and its derivatives, we report all iterations, including the steps rejected by the trust-region algorithm which will be responsible for “jumps” in the energy convergence in the figures. Our implementation is available at https://github.com/NGCartier/SCF-RDMFT_Hess_investigation and uses PySCF software^66^ and the C++ implementation of the fides package,^67^ relying on an algorithm proposed by Coleman and Li.^68,69^ This method is expensive because of the use of the eigenvalue decomposition of the Hessian. However, a trust-region conjugate-gradient method, which is much more affordable, could be used as a more economical alternative.

Throughout this work, we use the explicit by implicit (EBI) method^53^ to impose the *N*-representability conditions on the ONs. The EBI approach consists of parameterizing the ON , where *x*_*i*_ are the variables to optimize and μ is computed to satisfy the constraint on the trace of the 1-RDM. Since many functionals (including the Müller functional) depend on the square root of the ONs, in order to avoid diverging derivatives for *n*_*i*_ going to 0,^70^ we adapt the EBI parameterization as follows
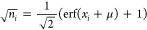
10

The NO orthonormality is imposed by a unitary parameterization via an exponential (*U* = exp(*X*)), as explained in the Introduction.

**Figure 1:**
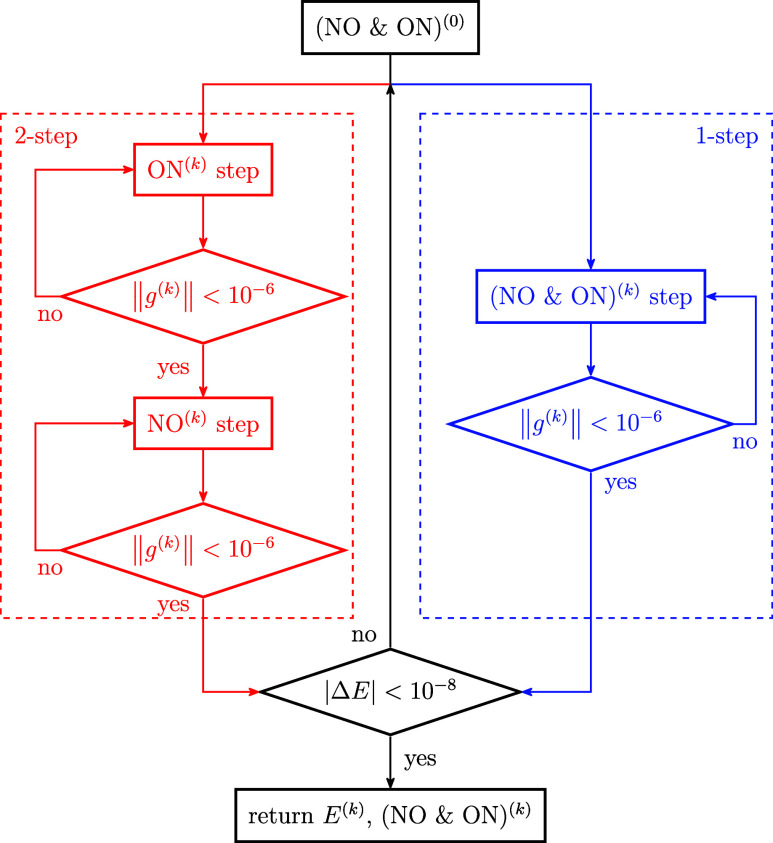
Algorithm of the 2-step procedure in red; modifications to obtain the 1-step version in blue and the common part in black.

We test our algorithm on alkanes, primary alcohols, and the H_2_O, HF, and N_2_ molecules. As an example of a strongly correlated system, we also add a stretched version of the N_2_ molecule, at a bond length *R* equal eight times the equilibrium bond length *R*_*e*_, denoted N_2_(*R* = 8*R*_*e*_). The results are obtained in a cc-pVDZ basis for H_2_O, alkanes, and primary alcohols and cc-pVTZ for HF, N_2_, and N_2_(*R* = 8*R*_*e*_), starting from Hartree–Fock orbitals and a Fermi–Dirac-like distribution of the ONs. For the microiterations (i.e., single update of the ONs or NOs), we used the gradient, ∥*g*^(*k*)^∥ < 10^–6^ (i.e., 2-norm of the gradient), as the termination criterion, and for the macroiterations, we used the energy difference with respect to the previous macroiteration, |Δ*E*| < 10^–8^ for both our implementation of 2-step and 1-step algorithms as depicted in Figure 1. To evaluate the convergence of the energy, we report at each microiteration the energy difference with the best energy *E*^ref^ we could obtain for each system.

## 3 Exact Hessian

In the following, we use Greek indices for the atomic orbital basis and Latin ones for the NO basis. Expanding the NOs in a given orbital basis {|χ_μ_⟩} naively as
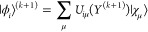
11(where *Y* is an antisymmetric matrix) leads to a very complicated form of the gradient and the Hessian since the derivatives of the unitary matrix *U* at general *Y* are very complicated. However, at *Y* = 0, the derivatives become quite simple,^57^ so instead, the expansion is only made about the current iterate
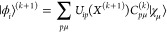
12where *C*^(*k*)^ = *U*(*X*^(*k*)^)*C*^(*k*–1)^ and *C*^(0)^ = *S*^–1/2^, with *S* as the overlap matrix (*S*_μν_ = ⟨χ_μ_|χ_ν_⟩). Note that exp(*X*^(*k*+1)^) = exp(*Y*^(*k*+1)^)exp(−*Y*^(*k*)^) ≠ exp(*Y*^(*k*+1)^ – *Y*^(*k*)^) as *Y*^(*k*+1)^ and *Y*^(*k*)^ do not commute in general. Expanding the derivatives around the current iterate is exact for an exact energy Hessian *H*^exa^, but in the case of an approximate Hessian, more care is needed as it is based on the difference between gradients at different iterates (see eq 39 in the Supporting Information). In the {|χ_μ_⟩} basis, the 1-RDM then attains the form
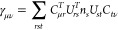
13

To derive the gradient and exact Hessian, *U*(*X*) can be expanded in a Taylor series for common parameterizations (Cayley,^71^ exponential). For simplicity and consistency with our implementation, we will take the case *U*(*X*) = exp(*X*); we have . The first and second derivatives are then
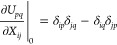
14a
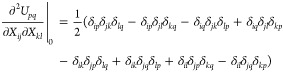
14b

Inserting it into the derivative of eq 13, we get
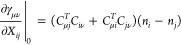
15a
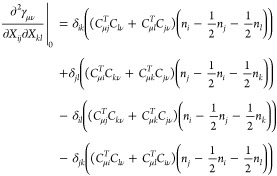
15b

Many 1-RDM functional approximations are explicitly written in the NO basis because of a dependence *F*(*n*_*i*_, *n*_*j*_) on two ONs.^24,45−48,52^ The energy functional then takes the form
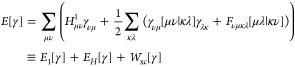
16where  and *F*_νμκλ_ = ∑_*ij*_*C*_ν*i*_^*T*^*C*_*i*μ_*F*(*n*_*i*_,*n*_*j*_)*C*_κ*j*_^*T*^*C*_*j*λ_, the approximated part of the functional.

From there, we can derive explicit expressions of the entries of *H*^exa^ (see Appendix A).

In the following, we will show that the exact Hessian provides a good convergence. Unfortunately, the excessive cost to compute *H*^exa^ of  (with *N*, number of orbitals) makes its use for practical calculations not a viable option. Therefore, we will aim for an efficient approximation that retains as much *H*^exa^ as possible in Sections 5 and 6.

## 4 Consistency between NOs and ONs

**Figure 2:**
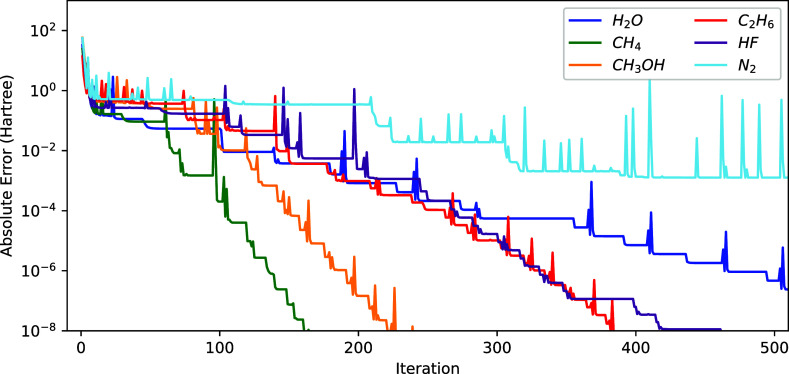
Convergence of the energy (*E*^(*k*)^ – *E*^(ref)^ with respect to the sum of NO and ON iteration) using the 2-step procedure (see text) for different molecules.

It is common in RDMFT to optimize the NOs and ONs successively in two separate steps. However, if not done carefully, a 2-step procedure may lead to a waste of computational time, when the implementation keeps optimizing the NOs to a high precision, though the change of the ONs in the next step will make such a high precision irrelevant (the reciprocal is also true, but the impaired computational cost is negligible). As a demonstration, we show the energy convergence at each consecutive microiteration step of a 2-step optimization in Figure 2. The wasteful NO optimization steps are reflected by the plateaus in the energy convergence. It is possible to limit this unwanted behavior by choosing, for example, a gradually tighter termination criterion for the optimization of the ONs and NOs, but the choice of such a procedure can be rather difficult (see Supporting Information Section 4 for details).

**Figure 3:**
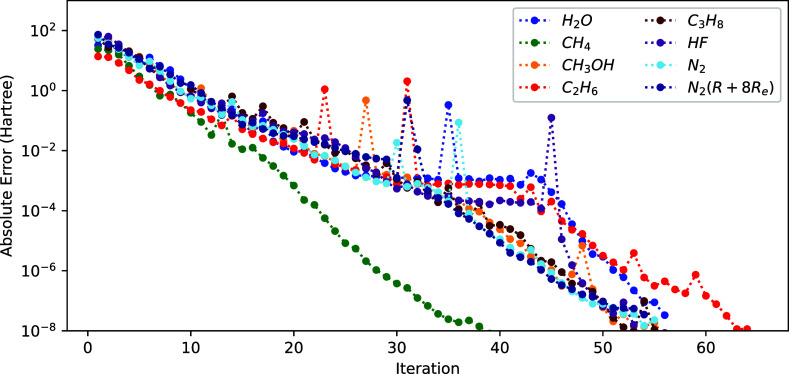
Convergence of the energy (*E*^(*k*)^ – *E*^(ref)^ with respect to the sum of NO and ON iteration) using *H*^exa^ for different molecules.

The problem of wasteful NO optimization steps is readily circumvented by optimizing both ONs and NOs simultaneously. An additional advantage is that the coupling between the ONs and NOs can readily be taken into account by including their cross derivatives in the Hessian. The results for this 1-step procedure based on the full exact Hessian are shown in Figure 3. The advantage of using the 1-step method is strikingly clear by comparing these results to the 2-step results from Figure 2. Indeed, we obtain a significant reduction of the total number of iterations, with the energy converging to an error of 5 × 10^–8^ Hartree for all molecules of the set within 70 iterations. The 2-step procedure, on the other hand, needs a few hundred iterations for the tested molecules. Moreover, the only additional cost per iteration for the 1-step procedure comes from the coupling between NOs and ONs, which is of order  only (we have *N* variables for the ONs). In contrast, the number of entries in the Hessian dedicated to NOs is of order  since the matrix parameterizing the NOs, *X*, counts *N*(*N* – 1)/2 degrees of freedom, for a basis of size *N*, which means that the cost of the coupling block is asymptotically negligible compared to the Hessian of the NOs.

For a fair comparison, we have tried to keep the implementations as similar as possible, so we have retained the macro-condition of the 2-step algorithm in the 1-step one. In principle, this condition should not play any role in the 1-step case as it acts as a double check by restarting from the converged point and does indeed not appear when using the exact Hessian. We, nonetheless, have observed that this restart of the 1-step optimization can regularly save the convergence as it resets the approximation of the Hessian, which may have significantly diverged from the exact one.

**Figure 4:**
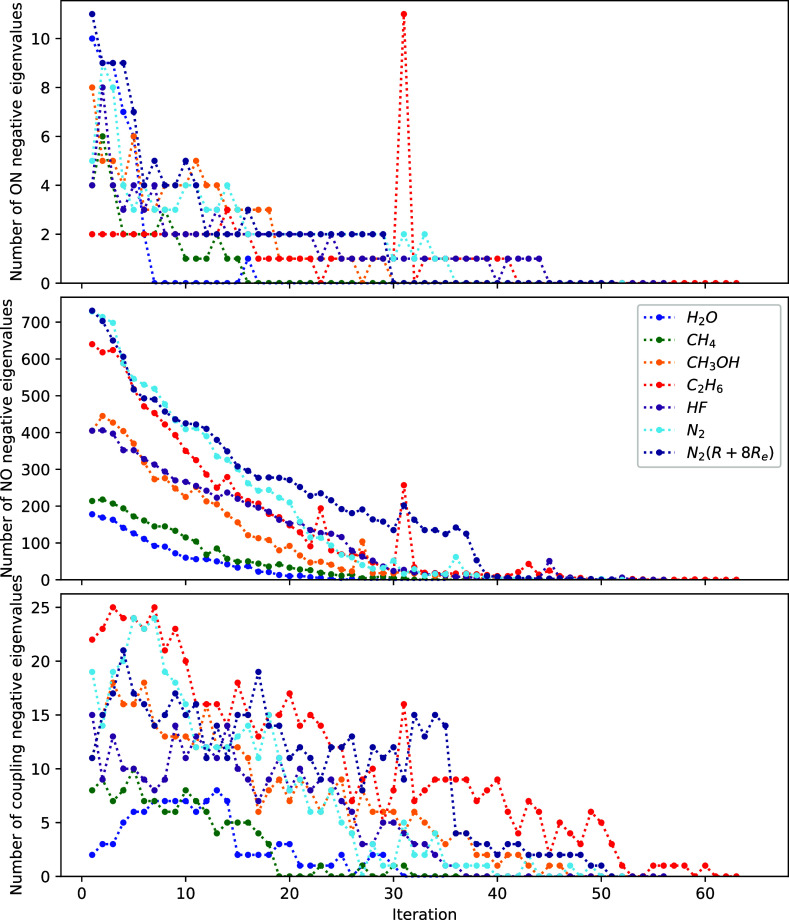
Number of negative eigenvalues of *H*^exa^, from the ON block (top panel) the NO block (middle panel), and the total number of negative eigenvalues minus the number of negative eigenvalues from the NO and ON blocks (bottom panel), for different molecules.

To verify that the 1-step algorithm converged to a minimum, we have computed the eigenvalues of *H*^exa^ and checked that they are all non-negative at the end of the convergence. We plot in Figure 4 the number of negative eigenvalues of the ON and NO block of the Hessian as well as the difference between the number of negative eigenvalues from the total Hessian and the two aforementioned blocks, which we can interpret as coming from the ON–NO coupling block. Indeed, the number of negative eigenvalues goes down to zero for all tested molecules. Note that the Hessian can initially contain a large number of negative eigenvalues, putting the emphasis on the nonconvexity of our problem even for the Müller functional. Moreover, most of the negative eigenvalues come from the NO block of the Hessian (middle panel), indicating that the hardest part to handle numerically is the optimization of the NOs.

**Figure 5:**
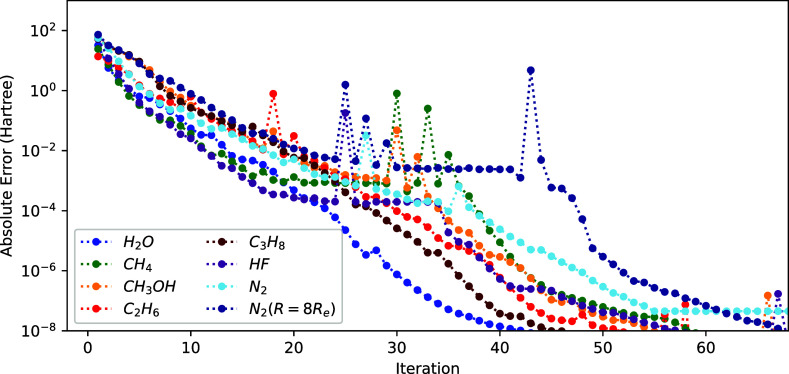
Convergence of the energy (*E*^(*k*)^ – *E*^(ref)^ with respect to microiteration count) using the exact ON–ON and NO–NO blocks of the Hessian and setting the ON–NO block to 0 for different molecules.

One may wonder how important the coupling block of the Hessian is, which we included in the 1-step procedure, especially as the number of negative eigenvalues (Figure 4) indicates that the most difficult part is actually the NO block. To investigate this point, we have tested the 1-step algorithm with the coupling block of the Hessian set to zero. The results are listed in Figure 5.

Comparing Figures 3 and 5, we observe that the convergences are roughly similar for most molecules, except for N_2_. Somewhat surprisingly, N_2_ at equilibrium distance is more challenging^a^ than stretched N_2_ which is typically considered to be more challenging due to its strong correlation character. The main difference between N_2_ with respect to the other systems is that some of the significantly fractionally occupied NOs are (nearly-)degenerate. It seems that the NO–ON coupling block is very helpful in handling those. However, for some systems (H_2_O, C_2_H_6_, and C_3_H_8_), setting the coupling block to zero yields faster convergence. Overall, it appears that the coupling block is important to make the 1-step method robust but does not affect the convergence rate very significantly. For an approximate Hessian, the coupling block seems to be somewhat more significant (see Supporting Information Section 1).

## 5 Extracting the Cheap Part of the Exact Hessian

**Figure 6:**
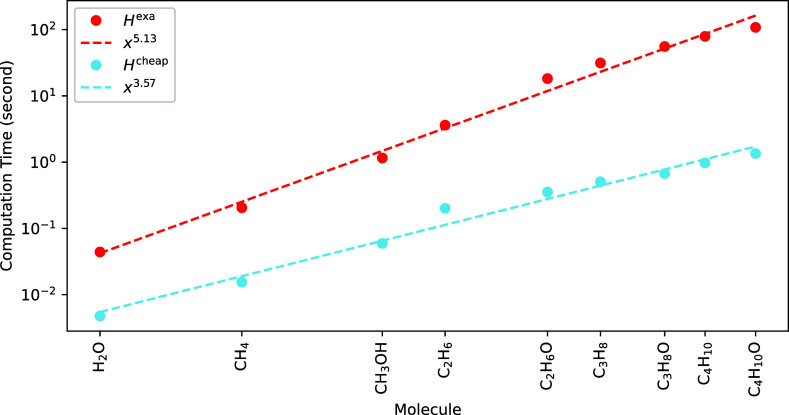
Computation time to compute the exact Hessian *H*^exa^ (red dots) and cheap part of the Hessian *H*^cheap^ (blue dots) with respect to the size of different molecules. Asymptotic behaviors are fitted (dashed lines), and the formal scaling is  for *H*^exa^ and  for *H*^cheap^.

For functionals of the form *F*(*n*_*i*_, *n*_*j*_) = *f*(*n*_*i*_)*f*(*n*_*j*_), sometimes called separable functionals, we can use the idea proposed by Giesbertz^72^ to reduce the scaling of the contribution to *H*^exa^ from the first term of eq 30, first two terms of eq 33, and the first height lines of eq 37, from  to  (see Figure 6). Indeed, denoting *v*_*ij*_^*K*^ = ∑_*p*_*f*(*n*_*p*_)[*ip*|*jp*], eqs 30, 33, and 37 become
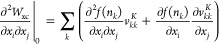
17
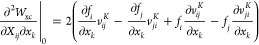
18and
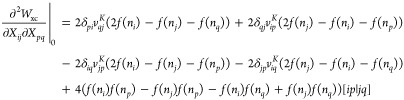
19

We can analogously compute the corresponding terms of eqs 29, 32 and 36 in , by computing separately *v*_*ij*_^*J*^ = ∑_*p*_*f*(*n*_*p*_)[*ij*|*pp*]. Moreover, the one-electron part of *H*^exa^ can clearly be obtained in  [even only ].

For the following expressions, we use uppercase for indices going from *N* + 1 to *N*(*N* + 1)/2, which can more conveniently be mapped to two indices (the first one going from 1 to *N* and the second one being strictly lower than the first one). We denote *H*^exp^ such that
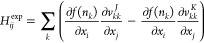
20afor the ON block
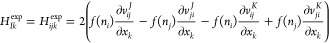
20bfor the coupling block and
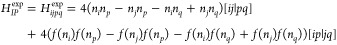
20cfor the NO block and *H*^cheap^ = *H*^exa^ – *H*^exp^. The scaling to obtain *H*^cheap^ is then  for a separable functional (that is, the same as to obtain the gradient), and we want to approximate *H*^exp^ only.

**Figure 7:**
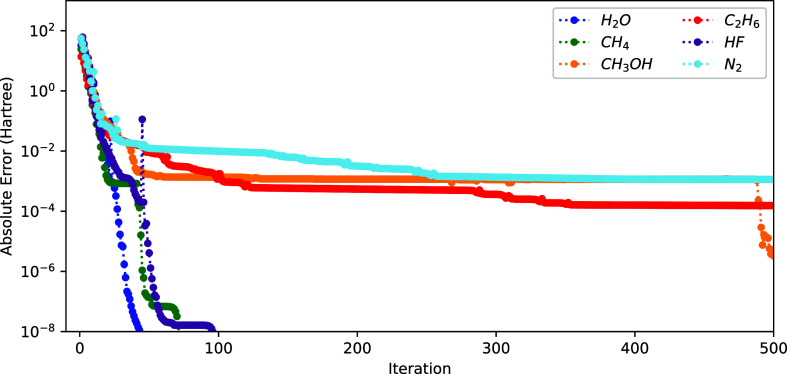
Convergence of the energy (*E*^(*k*)^ – *E*^(ref)^ with respect to microiteration count) using *B* = *H*^cheap^ for different molecules.

**Figure 8:**
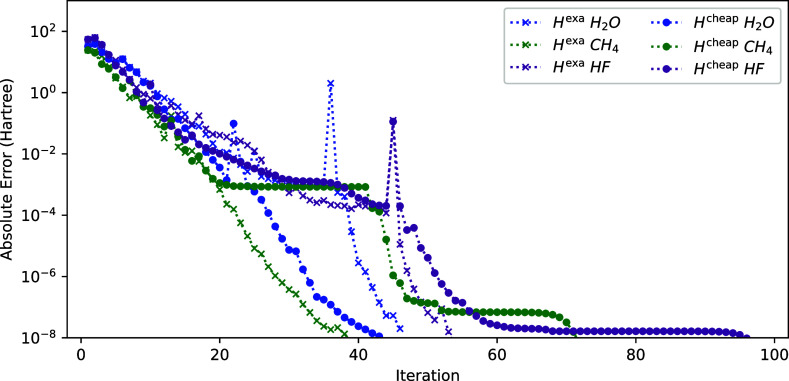
Comparison of the convergence of the energy (*E*^(*k*)^ – *E*^(ref)^ with respect to microiteration count) using *B* = *H*^cheap^, denoted by • and *B* = *H*^exa^, denoted by × for different molecules.

To do so, we can first neglect *H*^exp^ entirely and take *B* = *H*^cheap^ as a Hessian approximation. (We use *B* to denote an approximation to the Hessian). We tried it for our set of molecules in Figure 7 and observed good convergence for the first few tens of iterations. This approximation can even accidentally outperform the exact Hessian (see the H_2_O molecule in Figure 8). However, this is a very crude approximation, and the algorithm cannot converge for half of the molecules. This seems to indicate that *H*^cheap^ provides a good approximation of *H*^exa^ for the first few iterations which is sufficient to make H_2_O, CH_4_, and HF converge up to 10^–8^ Hartree but only 10^–2^ for N_2_ and 10^–4^ for C_2_H_6_.

**Figure 9:**
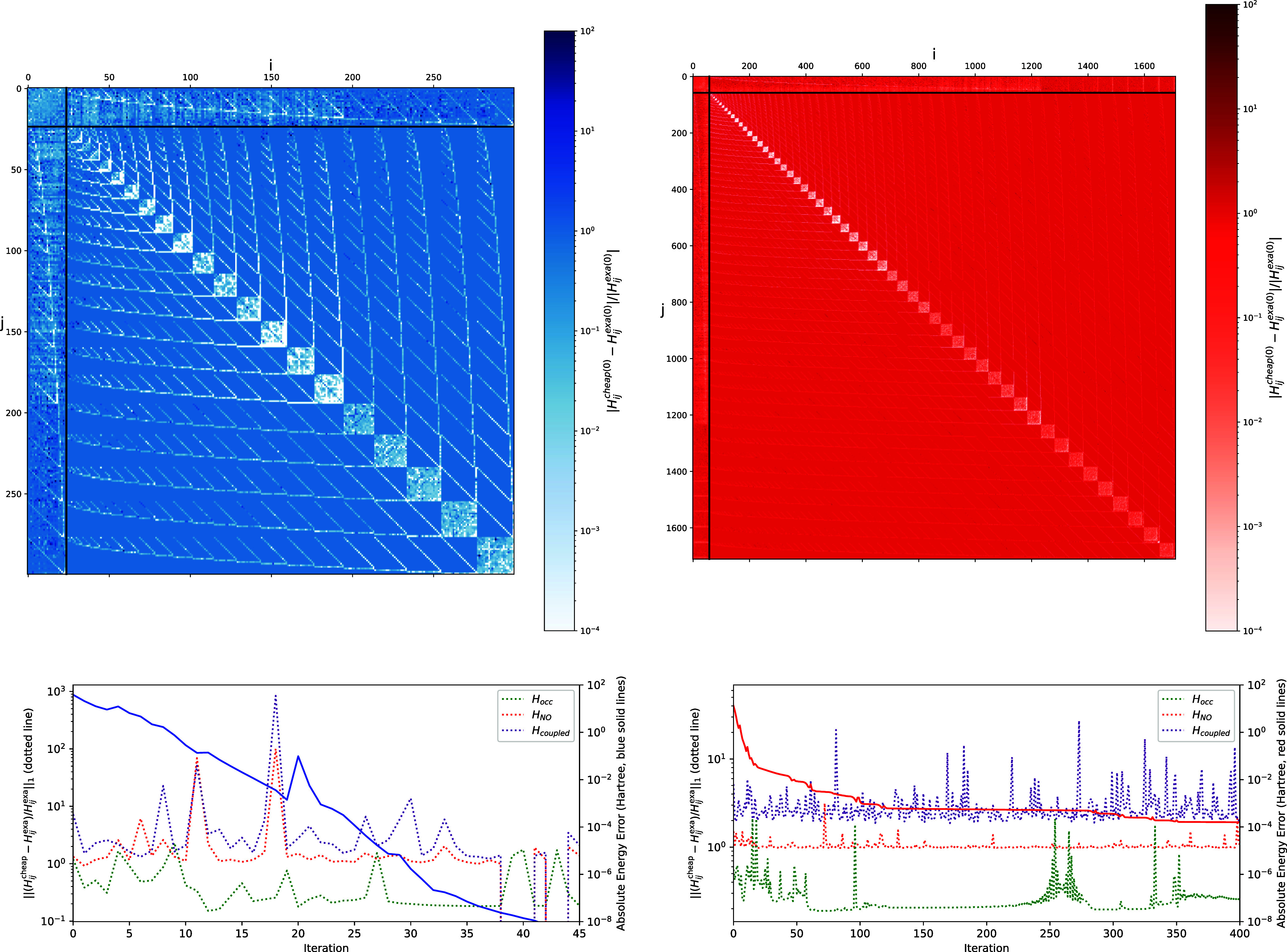
Relative error of *H*^cheap^ in the first iteration  (top panels); the black lines indicate the separation between the different blocks of the Hessian. And 1-norm of that relative error along the optimization  divided by the number of entries of the Hessian block (dotted lines in bottom panels) for the ON block (green lines), NO block (red lines), and coupling block (purple lines). Results are shown for the H_2_O (left panels) and C_2_H_6_ molecule (right panels).

To assess this assumption, we looked at the error made by taking *B* = *H*^cheap^ in Figure 9 for the different blocks of the Hessian. This shows that the ON block of the Hessian is decently approximated by *H*^cheap^, while the off-diagonal elements of the NO block and the coupling block are less accurate. The pattern of the upper panels of Figure 9 shows that the error in the NO block remains low when two indices of *X*_*ij*_ and *X*_*pq*_ are equal, that is, when *H*_*ijpq*_^cheap^ ≠ 0, highlighting the use of *H*^cheap^.

To avoid convergence issues for most systems, we thus need a suitable approximation of *H*^exp^ and will propose one in Section 6.

## 6 Approximate Hessians

The most common numerical approximation of the Hessian in the literature is the BFGS approximation.^73^ The BFGS update is obtained by demanding that the new approximate Hessian *B* is close to the previous one *B*^(*k*)^ under the constraints that the new approximate is symmetric and that *B* satisfies the secant equation.^73^ That is, the BFGS update solves
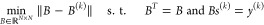
21where *s*^(*k*)^ and *y*^(*k*)^ are the step and difference of the gradient between the (*k* + 1)th and *k*th iterations, respectively. The constraint *Bs*^(*k*)^ = *y*^(*k*)^ is the secant equation, which simply means that the Hessian corresponds to a quadratic model through the last two iterates. Using the Frobenius norm, we obtain the standard BFGS update (see Section 6 of the Supporting Information for details)
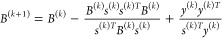
22

Unfortunately, the bare BFGS approximation of the Hessian does not provide a decent approximation of the exact Hessian, leading to poor convergence of the energy (see Section 2 of the Supporting Information).

The BFGS approximation is intended to approximate the Hessian for convex problems. However, due to the parameterization, we do the energy minimization with respect to ***x*** = (*x*, *X*^up^), where *X*^up^ is the vector of the strictly upper triangle entries of *X*. We will refer to the space of these variables as the ***x***-space.

On the other hand, we will call the **ν**-space (“NU-space”) the space where the energy derivatives can be directly calculated, i.e. , where *U* comprises all entries of the full *N* × *N* matrix. We can readily transform the relevant derivatives from the **ν**-to the ***x***-space using the Jacobian matrix  and its derivative. The Hessian tends to have a nicer expression in **ν**-space (is often quartic in *U* and the square roots of ONs), meaning that we can hope that the BFGS provides a better approximation in this space. Also, the fact that the unitary parameterization tends to make the Hessian indefinite indicates that the BFGS update on the Hessian in **ν**-space might work better.

Since we can readily calculate the cheap part of the Hessian in  (see Section 5), we only need to approximate the remaining part in **ν**-space as *B*_ν_^exp^ ≈ *H*_ν_^exp^.

Denoting ∇_**ν**_, the gradient in **ν**-space, and ∇_**x**_^2^, the Hessian operator in ***x***-space, the expensive energy Hessians *H*^exp^ in ***x***- and **ν**-space are related by the chain rule as
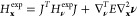
23and the total Hessian is
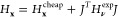
24

Now we have several choices to define the closeness to the previous approximation. One sensible option (another is presented in the Supporting Information in Section 3.2) is to make the Hessians close in **ν**-space while still demanding the secant equation in ***x***-space, so
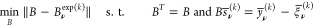
25where
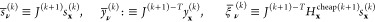
26

We thus only have to replace *B* → *B*_ν_^exp^,  and  in eq 22 and obtain
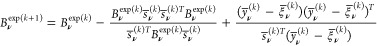
27

**Figure 10:**
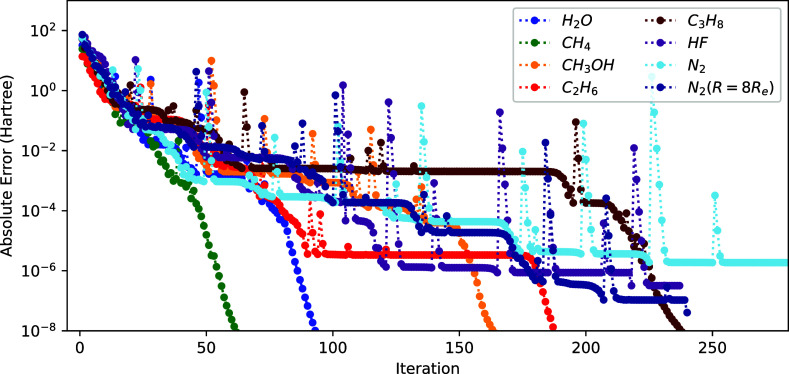
Convergence of the energy (*E*^(*k*)^ – *E*^(ref)^ with respect to microiteration count) using *H*_**x**_^cheap^ exactly and eq 27 to approximate *H*^exp^.

This approximation (Figure 10) unfortunately cannot match the good behavior of the *B*_**x**_ = *H*_**x**_^cheap^ approximation (Figure 7) for the first iterations. In Section 5, we attribute the initial good convergence of the *B*_**x**_ = *H*_**x**_^cheap^ approximation to a sufficient approximation of the Hessian. To take full advantage of the good convergence of *B*_**x**_^exp^ = 0, we then decided to turn on the BFGS approximation of *H*^exp^ only when the algorithm started to converge with *B*_**x**_ = *H*_**x**_^cheap^ to correct the convergence if it was not going to an actual minimum. Since *H*_**x**_^cheap^ approximated the ON block of the Hessian particularly well, it seems reasonable to look primarily at the ON step. In practice, we add a factor  to *B*_**x**_^exp^, where |*s*_*n*_|_*∞*_ is the *∞*-norm of the ON step.

**Figure 11:**
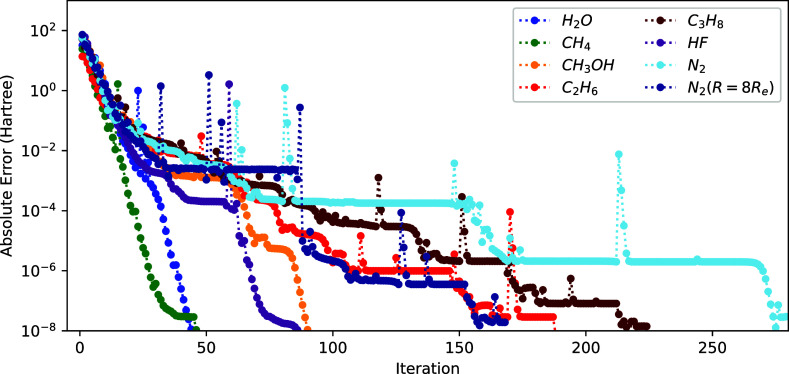
Convergence of the energy (*E*^(*k*)^ – *E*^(ref)^ with respect to microiteration count) using *H*_**x**_^cheap^ exactly and eq 27 to approximate *H*_**x**_^exp^ with the prefactor *A*(*s*_*n*_, 10^–3^) (see text).

We report the results in Figure 11. By comparing Figures 10 and 11, we see that prefactor *A* significantly improves the convergence, even beyond the first few iterations. Although the convergence is not as good as for the exact Hessian (Figure 3), it is able to reach an error of 2 × 10^–8^ Hartree within 280 iterations for all molecules. To reach 10^–8^ Hartree, we would need to use a termination condition stronger than our default settings (see Figure 1).

**Figure 12:**
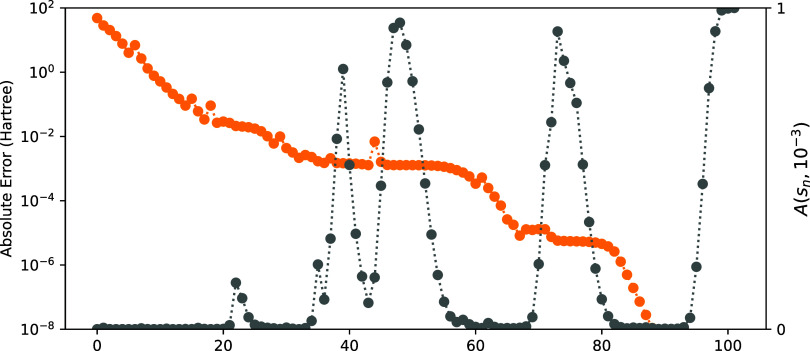
Absolute energy error (orange line) and the prefactor *A*(*s*_*n*_, 10^–3^) used on *B*^exp^ in **ν**-space (gray line) with respect to the iteration during the SCF procedure, using eq 27, for the CH_3_OH molecule.

It may be informative to look at the evolution of *A* to understand the plateaus in the convergence of the energy for some molecules using *B*_ν_^exp^. We plot *A* and the energy error as functions of iteration in Figure 12. Only the result for the CH_3_OH molecule is reported here, but the results are qualitatively similar for the other molecules that do not converge with the *B*^exp^ = 0 approximation. We observe that *A* does go from 0 to 1, but oscillates, inducing the plateaus in Figure 11. This indicates that the choice of *A* is not optimal and that the algorithm can be improved by building a better prefactor.

In order to compare the above approximation with existing results, we tested our molecules with the Müller functional implemented in the natural orbital functional (NOF) theory module^74^ of the molgw software.^75^ This module is based on the widely used RDMFT program, DoNOF.^52^ DoNOF was developed for recent Piris NOF functionals,^24,47,48^ and it inherits the partitioning of the orbitals in seniority-zero subspaces, meaning that the “Müller” functional in this module does have to comply with additional constraints but is believed to be decently close to the actual Müller functional.

More precisely, DoNOF employs a 2-step procedure, optimizing the ONs with a limited memory BFGS approximation and the NOs by using an iterative diagonalization approach (with a maximum of 30 NO iterations per macroiteration). The latter corresponds to the steepest descent and thus struggles to converge to high precision. For that reason, it has been decided to report the results to reach an error of 10^–3^ Hartree (instead of 10^–8^ up to now) in Table 1. The DoNOF implementation does (exactly or with reduction of identity) the 4-index transformation explicitly, which needs to be computed at each NO iteration but only at the first ON iteration of a macroiteration, meaning that the total number of expensive iterations is equal to the number of NO iterations plus the number of macroiterations. On the contrary, if it would use the idea of ref (72), to avoid explicit 4-index transformations, the cost of NO and ON iterations would be of the same order and the total number of expensive iterations and the sum of the NO and ON iterations. We thus report in Table 1 the number of macro-, NO, and ON iterations. To compare to our code, we will focus on the number of expensive iterations as implemented in DoNOF (i.e., macro- plus NO iterations). We obtain that DoNOF needs between 3000 and 7000 iterations, while our algorithm always reaches the required error within 30 to 90 iterations, which is an improvement of 2 orders of magnitude.

**Table 1:** Comparison Between the Number of Iterations of DoNOF and Our Results of Figure 11^a^

	DoNOF			results of Figure 11
molecule	macro-iter.	NO iter.	ON iter.	iter.
H_2_O	96	2851	2266	28
CH_4_	104	3091	3186	19
CH_3_OH	185	5521	8035	59
C_2_H_6_	181	5401	17,553	62
HF	219	6541	12,581	36
N_2_	150	4471	13,424	61
N_2_(*R* = 8*R*_*e*_)	170	5071	13,123	87

a Number of iterations for the molgw implementation^74,75^ of the DoNOF code^52^ and our last approximation (Figure 11) to converge to an energy error of 10^–3^ Hartree for different molecules (the reference energy for the DoNOF code is the energy obtained after a large number of 200–300 iterations and, for our code, the energy obtained with the exact Hessian in Figure 3). For DoNOF, we report the number of macroiterations (left column), the total number of NO iterations (middle column), and the total number of ON iterations (right column).

## 7 Conclusions

In this work, we used an optimization based on the Hessian to reduce the number of iterations of the SCF-RDMFT procedure. We first added the cross-terms in the Hessian, providing an efficient 1-step optimization method, in contrast to the more common 2-step one. As a baseline, we have shown that trust-region optimization with the exact Hessian provides a very effective algorithm for this. However, using the exact Hessian makes the iterations too expensive to be useful in practice. Thus, we have derived an accurate approximation of the Hessian. We first extracted an affordable part of the exact Hessian. Using only this cheap part, the number of iterations is actually of similar order as for the exact Hessian if it converged, but half of our test set actually did not converge. A rather robust and effective approximation was obtained by using the cheap part of the Hessian exactly and using a BFGS-like approximation to only approximate the expensive part in the more suitable **ν**-space. This approximate Hessian resulted in an algorithm that converged for all eight molecules within a few hundred iterations to an error of less than 10^–8^ Hartree.

Although the numerical results show a great amelioration of the convergence compared with a naive algorithm, there is still some room for improvement compared with the exact Hessian. To enhance the convergence even further, a more accurate approximation of the Hessian seems to be required. A first step in this direction would be to build a consistent secant equation in ***x***-space.

The plateaus in the energy versus iteration probably indicate that the algorithm is struggling with relatively flat areas in the energy landscape, i.e., NOs close to 0 or 2, which may become especially problematic for large basis sets. This might also explain why, when using even the exact Hessian, convergence is not that fast as one would expect. So another direction to improve the algorithm is to avoid these flat areas, by modifying the parameterization.

We used only the Müller functional in our tests. Although the approximate Hessian presented here can be applied to nonseparable functionals, the computation of the energy for these functionals is already in  and using *H*^exa^ would therefore be preferable. Moreover, we can use the resolution of identity to obtain a scaling of  for both the computation of the energy and *H*^exa^. However, nonseparable RDMFT functionals are not convex and some even display discontinuities with respect to the occupations, which may hinder the convergence. The efficiency of our approach for nonseparable functionals has thus yet to be assessed, which will be the subject of future work.

## Appendix

### Expression of the Exact Hessian

The derivatives of the 1-RDM with respect to the *x*_*i*_ are obtained by simply taking the derivative of the NOs in eq 13. Likewise, we get the cross-derivative of the 1-RDM by taking the derivative of the NOs with respect to the *x*_*i*_ in eq 15a.

Inserting the derivatives of the 1-RDM in eq 16, we get, for the NO block of the Hessian

28

29

30

for the coupling block

31

32

if *F*(*n*_*i*_, *n*_*j*_) = *F*(*n*_*j*_, *n*_*i*_), which holds for all functionals we are aware of. And, for the ON block, we get

35

36

37

## Supporting Information Available

Additional Hessian approximations, termination criteria, and functionals (PDF)

## Footnotes

a The molecule N_2_ only converged to 3 × 10^–8^ Hartree in the first macroiterations which had 153 microcycles, and after stopping at the second macroiteration, it only converged to 1.2 × 10^–8^ Hartree.
