# Supplementary material for: Exploiting the Hessian for a Better Convergence of the SCF-RDMFT Procedure
Source: J Chem Theory Comput. 2024 Apr 26;20(9):3669–82. doi: 10.1021/acs.jctc.4c00118 (PMC11099978; doi:10.1021/acs.jctc.4c00118)
Supplement: Supplementary file 1 — ct4c00118_si_001.pdf [file ct4c00118_si_001.pdf]

Supporting Information: Additional Hessian Approximations, Termination Criteria and Functionals

Nicolas G. Cartier* and Klaas J. H. Giesbertz

Department of Chemistry & Pharmaceutical Sciences and Amsterdam Institute of Molecular and Life Sciences (AIMMS), Faculty of Science, Vrije Universiteit, 1081HV Amsterdam, The Netherlands

* E-mail: n.cartier@vu.nl

1 Relevance Of The Coupling Block Of The Approximate Hessian

In the article we compared the convergence with and without coupling block for the exact newton method (Section 4), and concluded that the coupling block did not significantly improved the convergence rate in that case: for some systems the energy converged faster, others slower. A more consistent improvement of the convergence rate is actually visible with an approximate Hessian. Here we tested the approximation eq 27, and can indeed see by comparing Figure 13 with 11 a faster convergence when adding the (approximate) coupling block. The plots not only display less plateaus, but it also takes less iterations for the algorithm to exit them (see H_2O, CH_3OH, HF and stretched N_2). A possible explanation is that the coupling block may help to determine early in the convergence which orbitals are almost fully or not occupied and avoid steps that would tend to make ONs too close to 0 and 2.

2 Convergence Of The BFGS Approximation

The BFGS approximation itself, given by eq 22, (see Sec. 6 for the derivation) does not provide a good convergence of the energy, as illustrated in Figure 14. The error induced by the BFGS approximation is actually large, as can be seen from Figure 15, where we plotted the error made by the BFGS approximation compared to the exact Hessian. Since the BFGS approximation encourages a positive Hessian it has a hard time reproducing correctly the exact Hessian (which is indefinite, see Figure 4) for the first few iterations, but is supposed to reduce the error while getting closer to the minimum. However, we observe that this is not the case, indicating that the BFGS approximation does not suffice to efficiently approximate the exact hessian. This motivated us to obtain a better approximation of the Hessian in the article.

3 Alternative BFGS Approximations

3.1 Approximation eq 27 for the total Hessian

In the article we investigated a modified BFGS approximation eq 27, applied to the expensive part of the Hessian H^{exp} only. Note that a similar approximation can be derived for the total Hessian too.

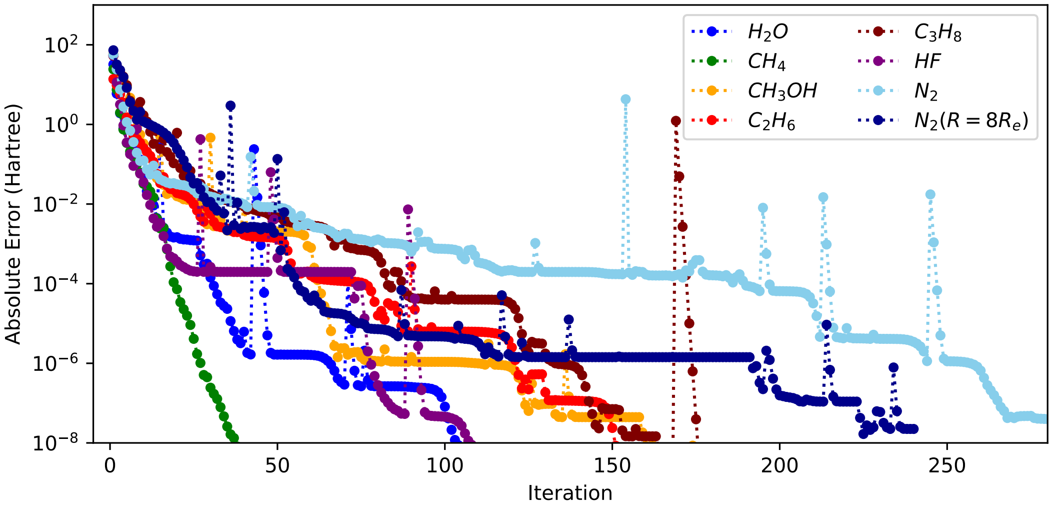

Figure 13: Convergence of the energy ($E^{(k)} - E^{(\mathrm{ref})}$ with respect to microiteration count) using $H_{\boldsymbol{x}}^{\mathrm{cheap}}$ exactly and eq 27 to approximate $H_{\boldsymbol{x}}^{\mathrm{exp}}$ with the prefactor $A(s_n, 10^{-3})$ (see text in the article) for the ON-ON and NO-NO blocks of the Hessian and setting the ON-NO block to 0 for different molecules.

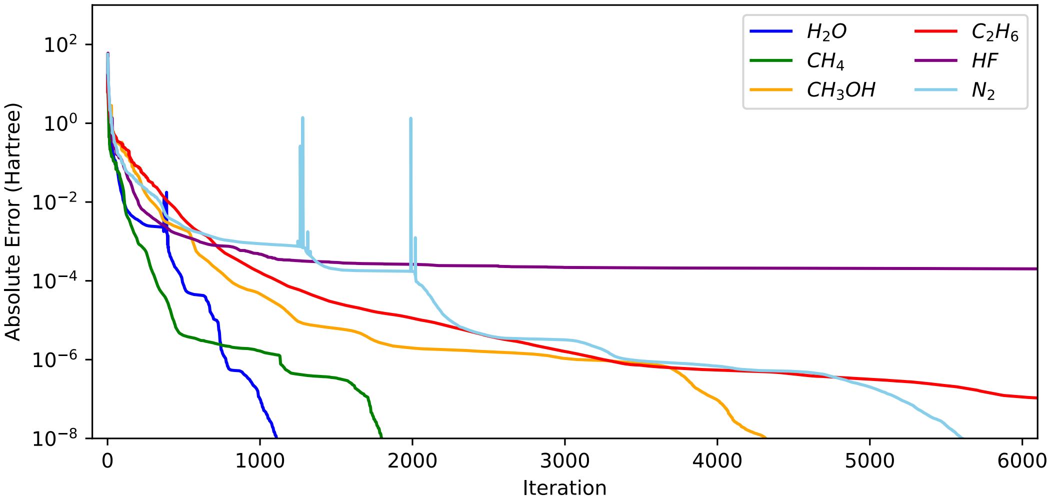

Figure 14: Convergence of the energy ($E^{(k)} - E^{(\mathrm{ref})}$ with respect to microiteration count) using a 1-step procedure with Hessian approximated by the BFGS method for different molecules.

Defining

$$\bar{\rho}_{\boldsymbol{\nu}}^{(k)} \equiv J^{(k+1)-T} \boldsymbol{\nabla}_{\boldsymbol{\nu}}^T E|_{(k+1)} \boldsymbol{\nabla}_{\boldsymbol{x}}^2 \boldsymbol{\nu}^{(k+1)} s_{\boldsymbol{x}}^{(k)}. \tag{38}$$

We simply need to set $y^{(k)} \to \bar{y}_{\boldsymbol{\nu}}^{(k)} - \bar{\rho}_{\boldsymbol{\nu}}^{(k)}$, $B^{(k)} \to B_{\boldsymbol{\nu}}^{(k)}$ and $s^{(k)} \to \bar{s}_{\boldsymbol{\nu}}^{(k)}$ in eq 22, which yields

$$B_{\boldsymbol{\nu}}^{(k+1)} = B_{\boldsymbol{\nu}}^{(k)} - \frac{B_{\boldsymbol{\nu}}^{(k)} \bar{s}_{\boldsymbol{\nu}}^{(k)} \bar{s}_{\boldsymbol{\nu}}^{(k)T} B_{\boldsymbol{\nu}}^{(k)}}{\bar{s}_{\boldsymbol{\nu}}^{(k)T} B_{\boldsymbol{\nu}}^{(k)} \bar{s}_{\boldsymbol{\nu}}^{(k)}} + \frac{\left(\bar{y}_{\boldsymbol{\nu}}^{(k)} - \bar{\rho}_{\boldsymbol{\nu}}^{(k)}\right)\left(\bar{y}_{\boldsymbol{\nu}}^{(k)} - \bar{\rho}_{\boldsymbol{\nu}}^{(k)}\right)^T}{\bar{s}_{\boldsymbol{\nu}}^{(k)T}\left(\bar{y}_{\boldsymbol{\nu}}^{(k)} - \bar{\rho}_{\boldsymbol{\nu}}^{(k)}\right)}, \tag{39a}$$

and transformed to $\boldsymbol{x}$-space via eq 23 becomes

$$B_{\boldsymbol{x}}^{(k+1)} = \tilde{B}_{\boldsymbol{x}}^{(k)} - \frac{\bar{B}_{\boldsymbol{x}}^{(k)} s_{\boldsymbol{x}}^{(k)} s_{\boldsymbol{x}}^{(k)T} \bar{B}_{\boldsymbol{x}}^{(k)}}{s_{\boldsymbol{x}}^{(k)T} \bar{B}_{\boldsymbol{x}}^{(k)} s_{\boldsymbol{x}}^{(k)}} + \frac{\left(y_{\boldsymbol{x}}^{(k)} - \rho_{\boldsymbol{x}}^{(k)}\right)\left(y_{\boldsymbol{x}}^{(k)} - \rho_{\boldsymbol{x}}^{(k)}\right)^T}{s_{\boldsymbol{x}}^{(k)T}\left(y_{\boldsymbol{x}}^{(k)} - \rho_{\boldsymbol{x}}^{(k)}\right)} \tag{39b}$$

where $\rho_{\boldsymbol{x}}^{(k)} \equiv J^{(k+1)T} \bar{\rho}_{\boldsymbol{\nu}}^{(k)} = \boldsymbol{\nabla}_{\boldsymbol{\nu}}^T E|_{(k+1)} \boldsymbol{\nabla}_{\boldsymbol{x}}^2 \boldsymbol{\nu}^{(k+1)} s_{\boldsymbol{x}}^{(k)}$, $\bar{B}_{\boldsymbol{x}}^{(k)} = J^{(k+1)T} B_{\boldsymbol{\nu}}^{(k)} J^{(k+1)}$.

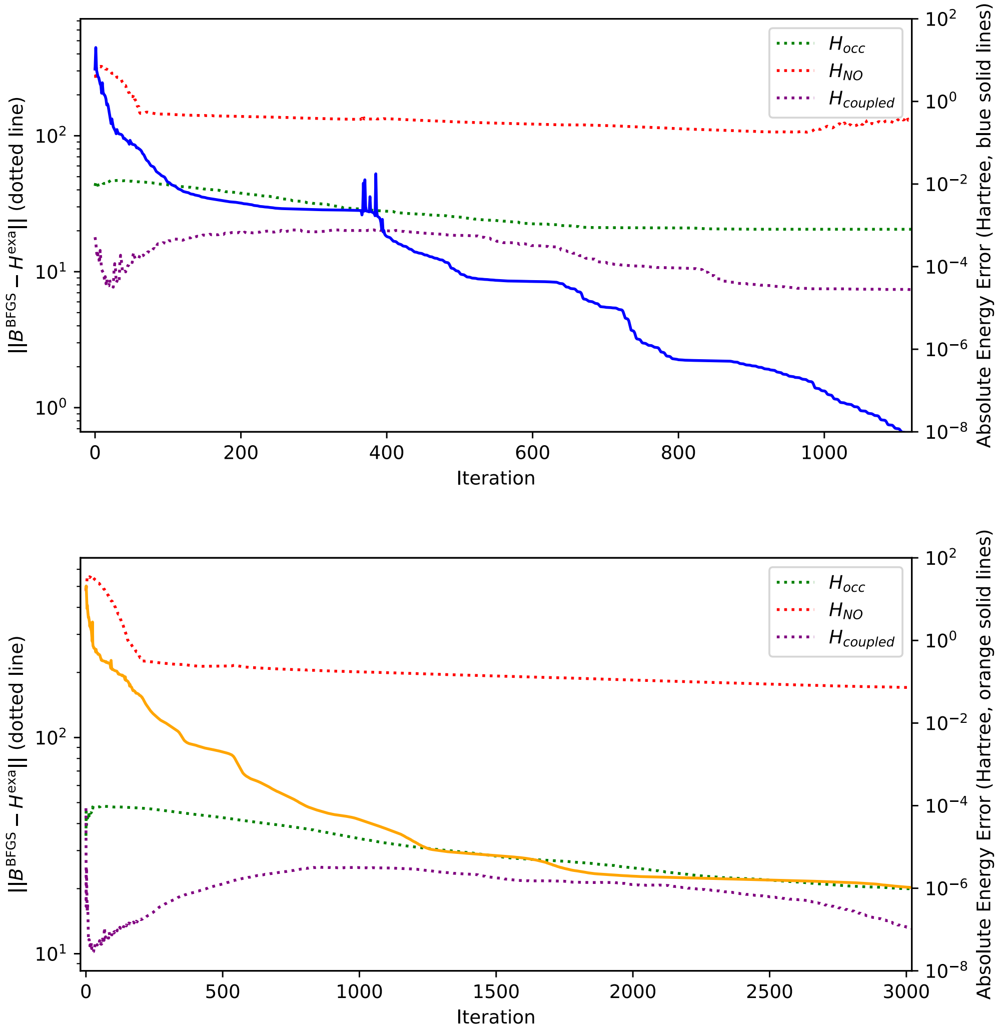

Figure 15: Error of the BFGS approximation $\|B^{\mathrm{BFGS}} - H^{\mathrm{exa}}\|$ (dotted line) for the H_2O molecule (top) and CH_3OH molecule (bottom). The error coming from the ON block is plotted as dotted red lines, from the NO block as green lines and from the coupling blocks as purple lines.

3.2 BFGS with closeness in $\boldsymbol{x}$-space

We focused, in the article on a modified BFGS approximation eq 27, based on the closeness in $\boldsymbol{\nu}$-space. An other option is to make the update close to the previous Hessian with updated Jacobian terms

$$\tilde{B}_{\boldsymbol{x}}^{(k)} \equiv J^{(k+1)T} B_{\boldsymbol{\nu}}^{(k)} J^{(k+1)} + \boldsymbol{\nabla}_{\boldsymbol{\nu}}^T E|_{(k+1)} \boldsymbol{\nabla}_{\boldsymbol{x}}^2 \boldsymbol{\nu}^{(k+1)}. \tag{40}$$

The BFGS optimization problem now becomes

$$\min_B \left\|B - \tilde{B}_{\boldsymbol{x}}^{(k)}\right\| \quad \text{s.t.} \quad B^T = B \text{ and } B s_{\boldsymbol{x}}^{(k)} = y_{\boldsymbol{x}}^{(k)}, \tag{41}$$

where $s_{\boldsymbol{x}}^{(k)}$, $y_{\boldsymbol{x}}^{(k)}$ are the step and difference of gradient between the $(k+1)^{th}$ and k^{th} iterations in $\boldsymbol{x}$-space. So we simply need to set $y^{(k)} \to y_{\boldsymbol{x}}^{(k)}$, $B^{(k)} \to \tilde{B}_{\boldsymbol{x}}^{(k)}$ and $s^{(k)} \to s_{\boldsymbol{x}}^{(k)}$, which yields the update

$$B_{\boldsymbol{x}}^{(k+1)} = \tilde{B}_{\boldsymbol{x}}^{(k)} - \frac{\tilde{B}_{\boldsymbol{x}}^{(k)} s_{\boldsymbol{x}}^{(k)} s_{\boldsymbol{x}}^{(k)T} \tilde{B}_{\boldsymbol{x}}^{(k)}}{s_{\boldsymbol{x}}^{(k)T} \tilde{B}_{\boldsymbol{x}}^{(k)} s_{\boldsymbol{x}}^{(k)}} + \frac{y_{\boldsymbol{x}}^{(k)} y_{\boldsymbol{x}}^{(k)T}}{y_{\boldsymbol{x}}^{(k)T} s_{\boldsymbol{x}}^{(k)}}, \tag{42a}$$

which can be transformed to the update in $\boldsymbol{\nu}$-space via the chain rule,

$$B_{\boldsymbol{\nu}}^{(k+1)} = B_{\boldsymbol{\nu}}^{(k)} + \frac{\bar{y}_{\boldsymbol{\nu}}^{(k)} \bar{y}_{\boldsymbol{\nu}}^{(k)T}}{\bar{y}_{\boldsymbol{\nu}}^{(k)T} \bar{s}_{\boldsymbol{\nu}}^{(k)}} - \frac{\left(B_{\boldsymbol{\nu}}^{(k)} \bar{s}_{\boldsymbol{\nu}}^{(k)} + \bar{\rho}_{\boldsymbol{\nu}}^{(k)}\right)\left(B_{\boldsymbol{\nu}}^{(k)} \bar{s}_{\boldsymbol{\nu}}^{(k)} + \bar{\rho}_{\boldsymbol{\nu}}^{(k)}\right)^T}{\bar{s}_{\boldsymbol{\nu}}^{(k)T}\left(B_{\boldsymbol{\nu}}^{(k)} \bar{s}_{\boldsymbol{\nu}}^{(k)} + \bar{\rho}_{\boldsymbol{\nu}}^{(k)}\right)}. \tag{42b}$$

The improvement w.r.t. the BFGS in $\boldsymbol{x}$-space (dashed lines) is significant for most molecules of the test set (see Figure 16), but the algorithm shows difficulties to converge for the HF, CH_3OH and C_2H_6 molecules. Indeed, it spends hundreds of iterations at an error of $2 \cdot 10^{-4}$ Hartree before resuming the convergence for the HF molecule (purple line) and simply stops without finding the correct minimum for the other two molecules.

3.3 $B^{\exp}$ approximation with closeness in $\boldsymbol{x}$-space

As we restricted eq 39 to $B^{\exp}$ to obtain eq 27 in the article, we can make the updated Hessian close to the previous Hessian with updated Jacobian terms and H^{cheap}

$$\tilde{B}_{\boldsymbol{x}}^{\exp(k)} \equiv J^{(k+1)T} B_{\boldsymbol{\nu}}^{\exp(k)} J^{(k+1)} + H_{\boldsymbol{x}}^{\text{cheap}(k+1)} \tag{43}$$

to restrict eq 42b to $B^{\exp}$. We simply have to replace $\tilde{B}_{\boldsymbol{x}}^{(k)} \rightarrow \tilde{B}_{\boldsymbol{x}}^{\exp(k)}$ in eq 42b and $\bar{\rho}_{\boldsymbol{\nu}}^{(k)} \rightarrow \bar{\xi}_{\boldsymbol{\nu}}^{(k)}$ ($\bar{\xi}_{\boldsymbol{\nu}}$ being defined in eq 26a), to get the update

$$B_{\boldsymbol{\nu}}^{\exp(k+1)} = B_{\boldsymbol{\nu}}^{\exp(k)} + \frac{\bar{y}_{\boldsymbol{\nu}}^{(k)} \bar{y}_{\boldsymbol{\nu}}^{(k)T}}{\bar{y}_{\boldsymbol{\nu}}^{(k)T} \bar{s}_{\boldsymbol{\nu}}^{(k)}} - \frac{\left(B_{\boldsymbol{\nu}}^{\exp(k)} \bar{s}_{\boldsymbol{\nu}}^{(k)} + \bar{\xi}_{\boldsymbol{\nu}}^{(k)}\right)\left(B_{\boldsymbol{\nu}}^{\exp(k)} \bar{s}_{\boldsymbol{\nu}}^{(k)} + \bar{\xi}_{\boldsymbol{\nu}}^{(k)}\right)^T}{\bar{s}_{\boldsymbol{\nu}}^{(k)T}\left(B_{\boldsymbol{\nu}}^{\exp(k)} \bar{s}_{\boldsymbol{\nu}}^{(k)} + \bar{\xi}_{\boldsymbol{\nu}}^{(k)}\right)}. \tag{44}$$

We plot the convergence using this approximation, with a damping prefactor A, as described in Section 6 of the article, see Figure 17. The update eq 44 seems to be overall less efficient than eq 27, especially for small molecules (H_2O, CH_4 and HF), suggesting that the prefactor should be modified for this approximation. Yet, eq 27 is not systematically better (compare the convergences of N_2 and C_2H_6 between Figure 11 and 17).

3.4 BFGS with the secant equation in $\boldsymbol{\nu}$-space

It needs to be mentioned that we do not implement the gradient difference exactly as needed in BFGS-like updates (eq 27 and eq 42), since it reads in full

$$y_{\boldsymbol{x}}^{(k)} = J^{(k+1)}(0) \boldsymbol{\nabla}_{\boldsymbol{\nu}} E\big|_{(k+1)} - J^{(k+1)}\left(-s_{\boldsymbol{x}}^{(k)}\right) \boldsymbol{\nabla}_{\boldsymbol{\nu}} E\big|_{(k)}. \tag{45}$$

Indeed, the Jacobian is very tough to evaluate for the exponential parametrization for $X \neq 0$. As a pragmatic solution, assuming that the NO step, s_{NO} is small enough (in $\boldsymbol{x}$-space), we only take the zeroth order term from its Taylor expansion for the part parametrizing the NOs,

$$J^{(k+1)}(-\{s_{n\boldsymbol{x}}^{(k)}, s_{NO\boldsymbol{x}}^{(k)}\}) \approx J^{(k+1)}(-\{s_{n\boldsymbol{x}}^{(k)}, 0\}). \tag{46}$$

This problem can actually be avoided by applying the BFGS procedure in the $\boldsymbol{\nu}$-space. The BFGS update in $\boldsymbol{\nu}$-space is obtained by simply putting the $\boldsymbol{\nu}$-space quantities into eq 22, which yields

$$B_{\boldsymbol{\nu}}^{(k+1)} = B_{\boldsymbol{\nu}}^{(k)} - \frac{B_{\boldsymbol{\nu}}^{(k)} s_{\boldsymbol{\nu}}^{(k)} s_{\boldsymbol{\nu}}^{(k)T} B_{\boldsymbol{\nu}}^{(k)}}{s_{\boldsymbol{\nu}}^{(k)T} B_{\boldsymbol{\nu}}^{(k)} s_{\boldsymbol{\nu}}^{(k)}} + \frac{y_{\boldsymbol{\nu}}^{(k)} y_{\boldsymbol{\nu}}^{(k)T}}{s_{\boldsymbol{\nu}}^{(k)T} y_{\boldsymbol{\nu}}^{(k)}}, \tag{47a}$$

which is then transformed to $\boldsymbol{x}$-space via the chain rule

$$B_{\boldsymbol{x}}^{(k+1)} = \tilde{B}_{\boldsymbol{x}}^{(k)} - \frac{\bar{B}_{\boldsymbol{\nu}}^{(k)} \bar{s}_{\boldsymbol{x}}^{(k)} \bar{s}_{\boldsymbol{x}}^{(k)T} \bar{B}_{\boldsymbol{\nu}}^{(k)}}{\bar{s}_{\boldsymbol{x}}^{(k)T} \bar{B}_{\boldsymbol{\nu}}^{(k)} \bar{s}_{\boldsymbol{x}}^{(k)}} + \frac{\bar{y}_{\boldsymbol{x}}^{(k)} \bar{y}_{\boldsymbol{x}}^{(k)T}}{\bar{s}_{\boldsymbol{x}}^{(k)T} \bar{y}_{\boldsymbol{x}}^{(k)}}, \tag{47b}$$

where $\bar{s}_{\boldsymbol{x}}^{(k)} \equiv J^{(k+1)-1} s_{\boldsymbol{\nu}}^{(k)}$ and $\bar{y}_{\boldsymbol{x}}^{(k)} \equiv J^{(k+1)} y_{\boldsymbol{\nu}}^{(k)}$. Though this update could be implemented without additional approximations, the BFGS update in the $\boldsymbol{\nu}$-space actually leads to more convergence problems than eq 42 as we demonstrate in Figure 18. We suspect that the is-

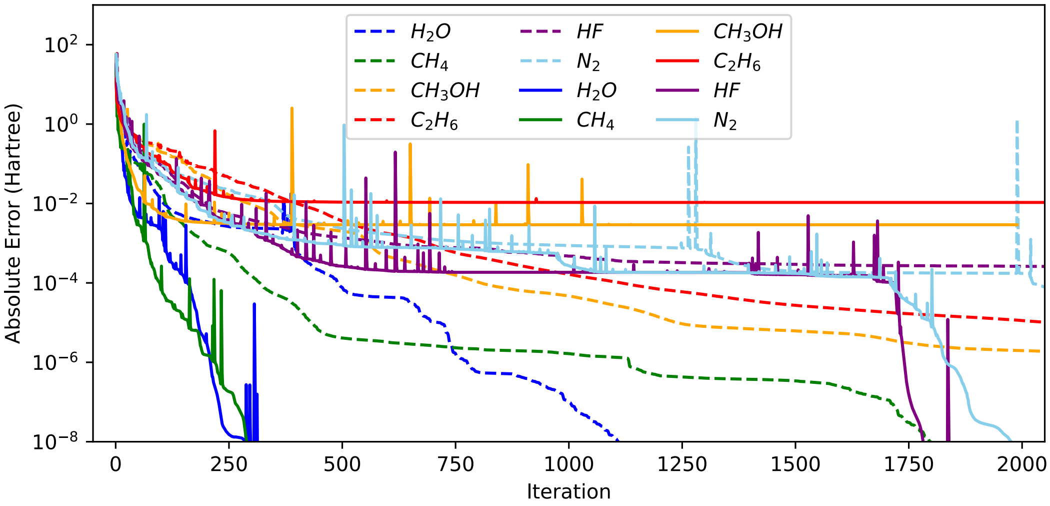

Figure 16: Convergence of the energy ($E^{(k)} - E^{(\text{ref})}$ with respect to microiteration count) using equation eq 42 in solid lines and eq 22 in dashed lines for different molecules.

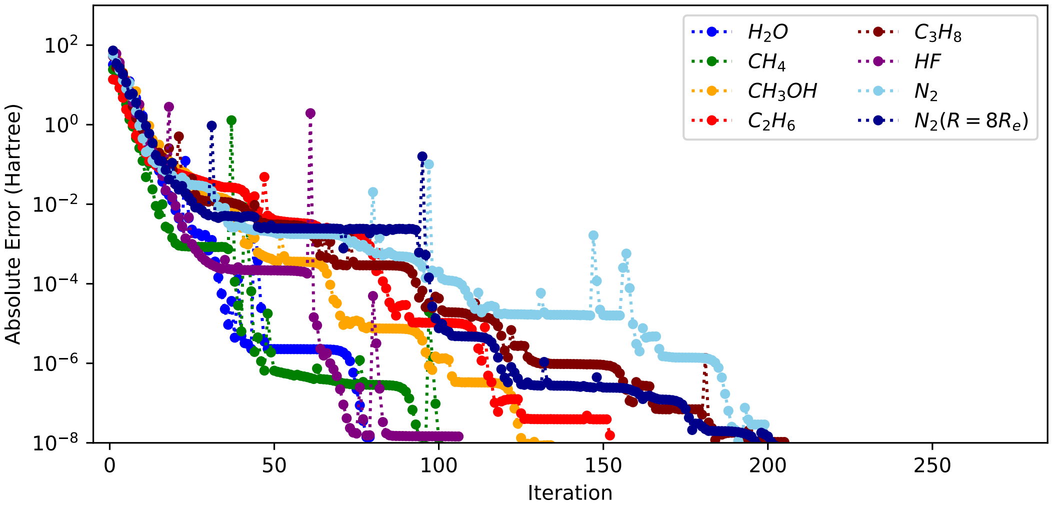

Figure 17: Convergence of the energy ($E^{(k)} - E^{(\text{ref})}$ with respect to microiteration count) using $H_{\boldsymbol{x}}^{\text{cheap}}$ exactly and eq 44 to approximate $H_{\boldsymbol{x}}^{\text{exp}}$ with the prefactor $A(s_n, 10^{-3})$ (see text in the article).

sue here is that the secant equation in $\boldsymbol{\nu}$-space is less relevant than the secant equation in $\boldsymbol{x}$-space, as we aim to make a quadratic model in $\boldsymbol{x}$-space.

4 Termination Criteria For The 2-Step Procedure

In the case of a 2-step procedure, to avoid the waste of NO iterations, a straightforward way would be to adapt the termination condition on the NO optimization while keeping a strong termination criterion on the global convergence. A criterion on the gradient seems more reasonable, yet to emphasis our point, in this section we take a termination condition on the step length $\|s_{NO}\| < \epsilon$,(where ϵ is the required termination criterion for one optimization of the NOs), which will exaggerate the problem. We present the results in Figure 19 and 20 with the exact Hessian and BFGS approximation respectively.

When used with a poor Hessian approximation (BFGS here) this approach leads to convergence problems (the darker blue lines in Figure 20 did not reach the macroiteration termination criterion), when the termination criterion on the

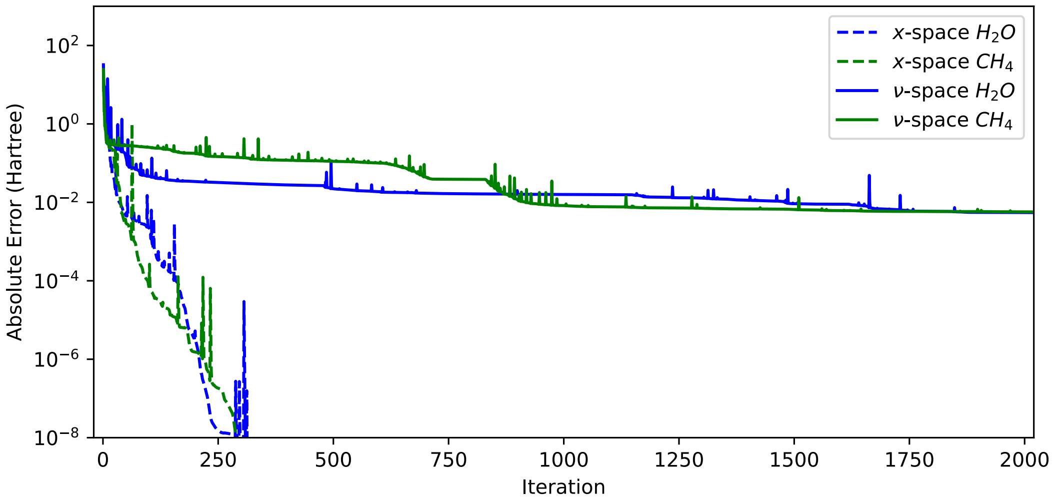

Figure 18: Convergence of the energy ($E^{(k)} - E^{(\mathrm{ref})}$ with respect to microiteration count) using eq 47 in solid lines and eq 42 in dashed lines for the $\mathrm{H_2O}$ and $\mathrm{CH_4}$ molecules.

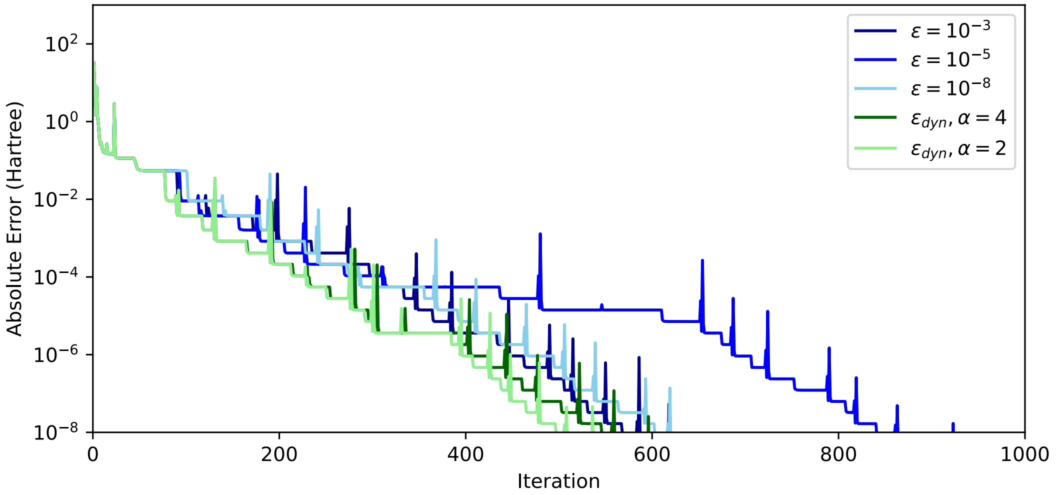

Figure 19: Convergence of the energy ($E^{(k)} - E^{(\mathrm{ref})}$ with respect to the sum of ON and NO microiteration counts) using different ϵ termination criteria for the optimization of the NO at each macroiteration, for the $\mathrm{H_2O}$ molecule, and exact Hessian. For ϵ_{dyn}, we start at $\epsilon_{\mathrm{dyn}}^{(0)} = 10^{-2}$ and take $\epsilon_{\mathrm{dyn}}^{(k+1)} = \frac{1}{\alpha}\epsilon_{\mathrm{dyn}}^{(k)}$ after each macroiteration until $\epsilon_{\mathrm{dyn}}^{(k)}$ reaches the same precision as the global convergence criterion.

NOs, ϵ is to loos, the algorithm fails to converge. On the other hand, a too strong criterion (i.e. small ϵ) forces the algorithm to compute many ineffective iterations (thousands of those in the lighter blue lines in Figure 20).

This problem is readily remedied by a dynamic termination condition for the termination threshold ϵ_{dyn} of the NO optimization. The dynamic NO termination criterion ϵ_{dyn} would start at a large value and be reduced (for example, after each macroiteration) to a sufficiently small value to ensure global convergence. As simple demonstration we have chosen the update $\epsilon^{(k+1)} = \epsilon^{(k)}/\alpha$. In Figure 19 and 20 we show the energy convergence for $\epsilon^{(0)} = 10^{-2}$ and both $\alpha = 2$ and $\alpha = 3$ in green. All values for α (green lines) are more efficient than the static NO convergence criteria (blue lines), especially for the BFGS case (Figure 20), but remain inefficient compared to a 1-step procedure (see dark blue line in Figure 3 and 14).

Another criterion that can be considered, is the number of iteration allowed per macroiteration.[1] The convergence is reported for some

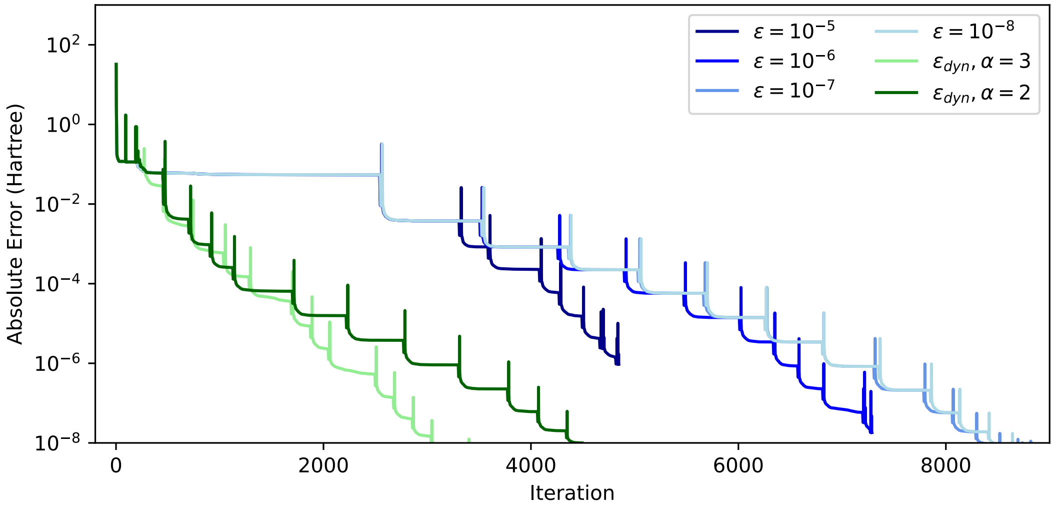

Figure 20: Convergence of the energy ($E^{(k)} - E^{(\mathrm{ref})}$ with respect to the sum of ON and NO microiteration counts) using different ϵ termination criteria for the optimization of the NO at each macroiteration, for the H_2O molecule, and BFGS as Hessian approximation. For ϵ_{dyn}, we start at $\epsilon_{\mathrm{dyn}}^{(0)} = 10^{-2}$ and take $\epsilon_{\mathrm{dyn}}^{(k+1)} = \frac{1}{\alpha}\epsilon_{\mathrm{dyn}}^{(k)}$ after each macroiteration until $\epsilon_{\mathrm{dyn}}^{(k)}$ reaches the same precision as the global convergence criterion.

set of maximum ON and NO iterations in Figure 21. We first notice that the number NO iteration cannot be to small, otherwise the update do not sufficiently change the energy and the algorithm stops (grey line). We can also notice that changing the maximum number of iteration can significantly impact the final number of iterations (purple line needs around 200 iterations to converge to 10^{-8} Hartree, while the red line, more than 400), and that a small number of NO iterations per ON iteration is more efficient. This indeed corresponds to limiting the amount of inefficient NO iterations.

5 Other Separable Functionals

5.1 The Power functional with $\alpha = 0.7$

Our approach requires additional work to be adapted to non-separable functionals, but can readily be employed for separable functionals other than Müller. Unfortunately, there are few of then, all taking the form,

$$W^{\mathrm{Power}}[\gamma] = \frac{1}{2}\sum_{ij} n_i n_j [ii|jj] - \frac{1}{2}\sum_{ij}(n_i n_j)^{\alpha}[ij|ji], \tag{48}$$

where $\alpha \in (0, 1]$. The Müller functional corresponds to the case $\alpha = 1/2$ (studied in the article), the Hatree–Fock functional (see Section 5.2), $\alpha = 1$, but other values of α have been proposed in the literature.[2–5] As an example, here we take $\alpha = 0.7$ and test our algorithm with the exact Hessian in Figure 22, approximations eq 27 and eq 44 (with H^{cheap} exactly and damping factor as in Section 6 of the article) in Figure 23 and Figure 24 respectively.
With the exact Hessian, the number of iterations to reach a precision of 10^{-8} Hartree is similar to the case of the Müller functional (see Figure 3 of the article) for most molecules, but the convergences display more unsuccessful steps (energy jumps) for diatomic molecules and C_3H_8, and fails to converge beyond an error of 10^{-7} (purple and light blue lines of Figure 22). The algorithm moreover struggles to converge for the strong correlated system (dark

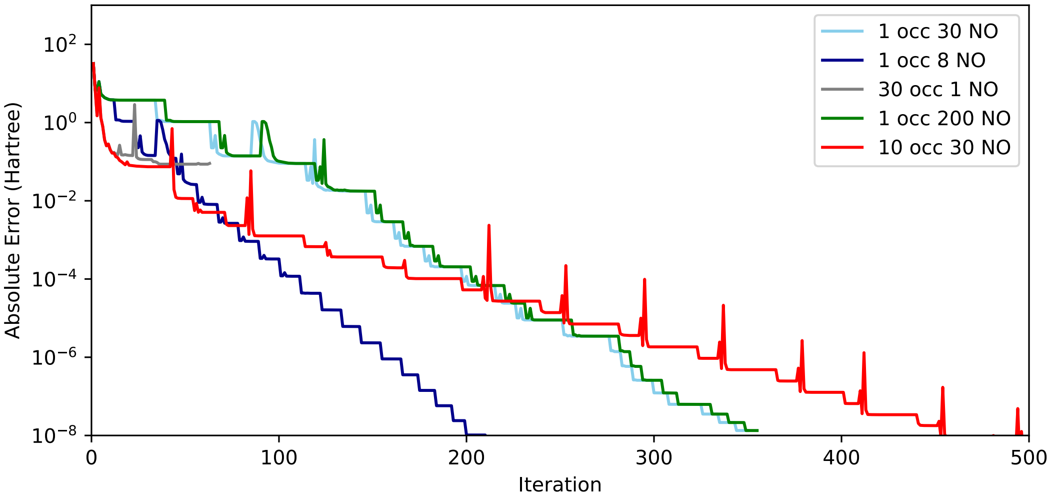

Figure 21: Convergence of the energy ($E^{(k)} - E^{(\mathrm{ref})}$ with respect to the sum of ON and NO microiteration counts) using different limits on the number of iteration for the optimization of the NO and ON at each macroiteration, for the H_2O molecule, and exact Hessian. The first number indicates the maximum number of ON iteration per macroiteration and the second the maximum number of NO per macroiteration.

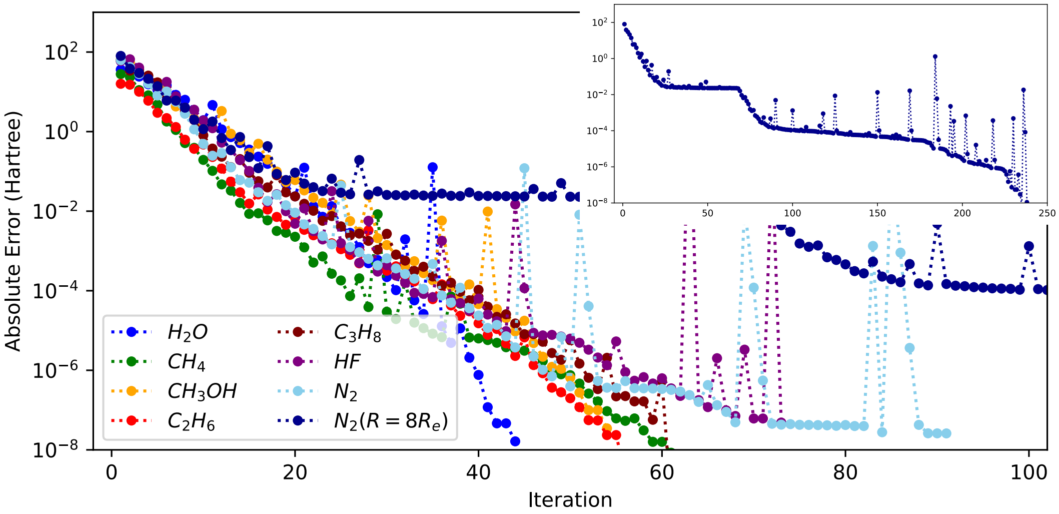

Figure 22: Convergence of the energy ($E^{(k)} - E^{(\mathrm{ref})}$ given by the Power functional ($\alpha = 0.7$) with respect to microiteration count) using the exact Hessian.

blue line), and only reaches the convergence criterion after 240 iterations.

The approximation eq 44 seems to work worse with the Power functional, it fails to converge for a certain number of molecules, including CH_4 which did not prove to be challenging up to this point (see Figure 23). Fortunately, the update eq 27 gives convergences comparable to the case of the Müller functional (Figure 24 compared to Figure 17), and most molecule reach an error of 10^{-7} to 10^{-8} Hartree (as the exact Hessian, Figure 22) within 200 iterations. A notable exception is the stretched N_2 molecule, which did not converge, but this system was already challenging for the exact Hessian.

5.2 The Hartree–Fock functional

Although, not practically relevant it is possible to define a functional in 1RDMFT, corresponding (i.e. giving the same ground state 1RDM and energy) to the Hartree–Fock method. On the contrary to the Müller functional, the Hartree–Fock is concave in the space

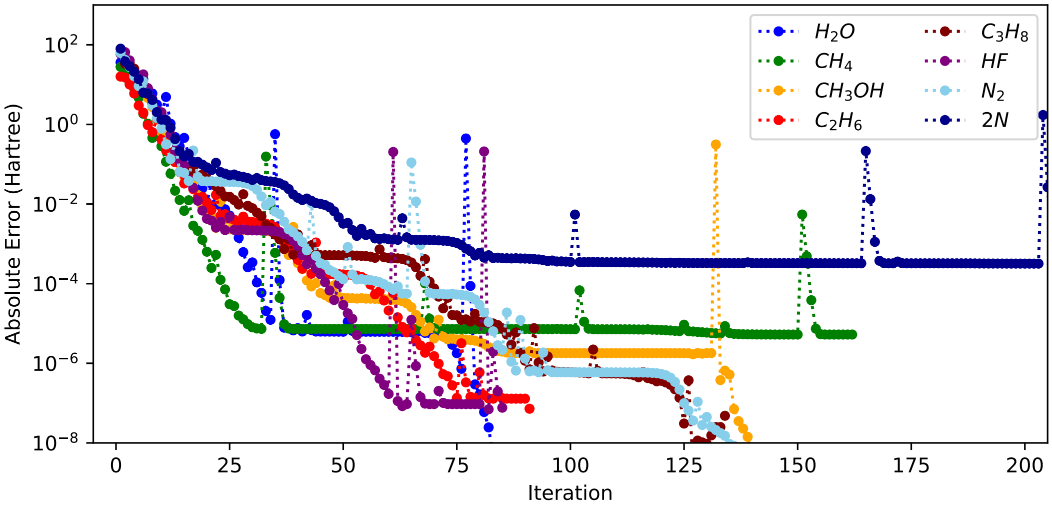

Figure 23: Convergence of the energy ($E^{(k)} - E^{(\mathrm{ref})}$ given by the Power functional ($\alpha = 0.7$) with respect to microiteration count) using $H_{\boldsymbol{x}}^{\mathrm{cheap}}$ exactly and equation eq 44 to approximate H^{exp} with the prefactor $A(s_n, 10^{-3})$ (see text in the article).

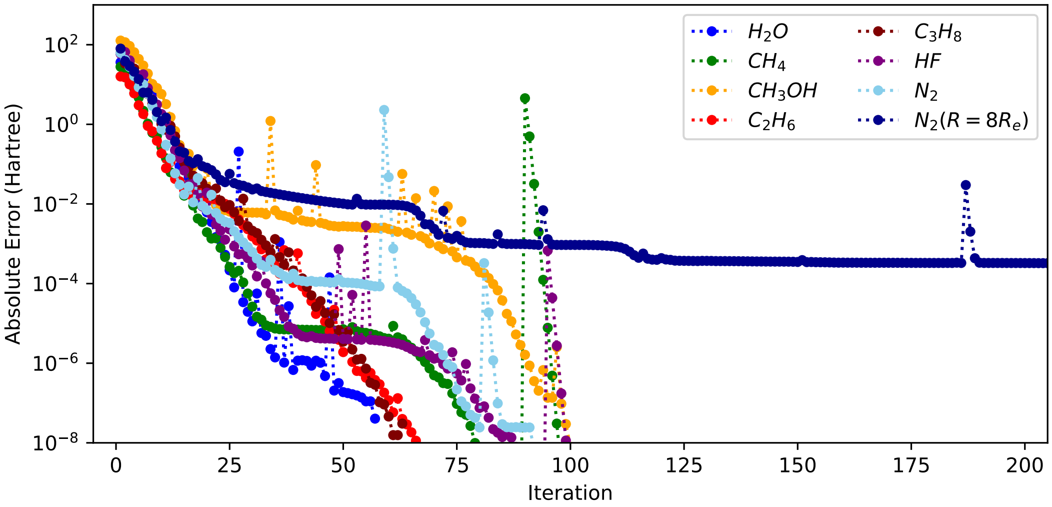

Figure 24: Convergence of the energy ($E^{(k)} - E^{(\mathrm{ref})}$ given by the Power functional ($\alpha = 0.7$) with respect to microiteration count) using $H_{\boldsymbol{x}}^{\mathrm{cheap}}$ exactly and equation eq 27 to approximate H^{exp} with the prefactor $A(s_n, 10^{-3})$ (see text in the article).

of 1RDM,[6,7] which makes it a relevant test for our method. We thus report the energy convergence with the Hartree–Fock functional when using the exact Hessian in Figure 25. While the algorithm converges quiet well to an error of 10^{-6} Hartree for all the tested molecules, it fails to improve beyond that precision. The reason is that the error function in the EBI parametrization tends to be very flat when the ONs tend to 0 or 2, which makes the gradient and Hessian vanish, preventing the algorithm from converging further. A way around it is to use an other parametrization associated with a projection of the ONs as proposed by Cancès and Pernal,[8]

$$\sqrt{n_i} = \begin{cases} \sqrt{2}\frac{x_i}{1+\nu} & \text{if } x_i \in [0, 1+\nu] \\ 0 & \text{if } x_i < 0 \\ \sqrt{2} & \text{if } x_i > 1+\nu, \end{cases} \tag{49}$$

where $x_i \in \mathbb{R}$ and $\nu \in \mathbb{R}^+$ s.t. $\mathrm{Tr}\{\gamma\} \leq N_e$. We have tested our algorithm while replacing only the way we handle constraints on the ONs i.e. EBI by eq 49 and report the results in Figure 26. Aside from some instabilities due to the imma-

turity of the code, the approach of equation eq 49 seems to indeed solve the problem and allow the algorithm to converge decently fast to 10^{-8} Hartree. It has also been possible to test the approximation of Section 6 of the article with the Hartree–Fock functional and using eq 49, which give a convergence comparable to the one of the exact Hessian (up to different jumps due to the instabilities), as shown in Figure 27.

6 Derivation Of The BFGS Approximation

The idea of (quasi-)Newton methods is to take the derivative of a second order model m of the function f to optimise, which writes for the kth iteration

$$\boldsymbol{\nabla} m^{(k)}(p) = \boldsymbol{\nabla} f^{(k)} + B^{(k)}p, \tag{50}$$

where B is the (approximate) Hessian. We then impose that the derivative of m coincides with the derivative of f at the last two iterations. For the last iterations it is automatically verified by taking $p = 0$, and for the second last iterations we have to take $p = -s^{(k-1)}$, that is, the opposite of the last step, and we get

$$\boldsymbol{\nabla} m^{(k)}(-s^{(k-1)}) = \boldsymbol{\nabla} f^{(k)} - B^{(k)}s^{(k-1)} = \boldsymbol{\nabla} f^{(k-1)}. \tag{51}$$

By defining $y^{(k-1)} = \boldsymbol{\nabla} f^{(k)} - \boldsymbol{\nabla} f^{(k-1)}$, we obtain the, so called, secant equation,

$$B^{(k+1)}s^{(k)} = y^{(k)}. \tag{52}$$

To obtain the BFGS update eq 22, we need to solve the problem eq 21

$$\min_{B \in \mathbb{R}^{N\times N}} \left\| B - B^{(k)} \right\| \quad \text{s.t.} \quad B^T = B \text{ and } Bs^{(k)} = y^{(k)}, \tag{53}$$

with the Frobenius norm for $\|\cdot\|$.
We follow the proof given by Hauser.[9] To ensure that B is symmetric, we write it as $B = LL^T$, $L \in \mathbb{R}^{N\times N}$. Taking $g = L^T s^{(k)}$ the secant equation becomes

$$Lg = y^{(k)}. \tag{54}$$

Taking the scalar product $\langle A|B\rangle = \mathrm{Tr}\left(AB^T\right)$, eq 53 is equivalent to

$$\min_{L} \left\langle L - L^{(k)} \middle| L - L^{(k)} \right\rangle \quad \text{s.t.} \quad \left\langle L \middle| e_i g^T \right\rangle = y_i^{(k)} \forall i, \tag{55}$$

with e_i the ith column of the identity matrix, that is $e_i g^T$ are the normal vectors defining the affine subspace $\mathcal{P}_g$ in which L has to be optimized. The minimizer L^* is the closest point to $L^{(k)}$ in $\mathcal{P}_g$. Thus, $L^* - L^{(k)}$ is orthogonal to $\mathcal{P}_g$ and so a linear combination of the $e_i g^T$. eq 53 is then equivalent to find $L^* \in \mathbb{R}^{N\times N}$ and $\lambda^* \in \mathbb{R}^N$ satisfying

$$(L^* - L^{(k)}) - \lambda^* g^T = 0 \tag{56a}$$

$$L^* g = y^{(k)}. \tag{56b}$$

Multiplying eq 56a from the right by g and utilizing eq 56b gives

$$\lambda^* = \frac{y^{(k)} - L^{(k)}g}{g^T g}, \tag{57a}$$

which can be inserted back to yield

$$L^* = L^{(k)} + \frac{(y^{(k)} - L^{(k)}g)g^T}{g^T g}. \tag{57b}$$

Inserting the minimizer L^* in the definition of g and rearranging gives

$$L^{(k)T}s^{(k)} = g\left(1 - \frac{(y^{(k)} - L^{(k)}g)^T s^{(k)}}{g^T g}\right), \tag{58}$$

meaning that $g \propto L^{(k)T}s^{(k)}$. We take $g = \beta L^{(k)T}s^{(k)}$ and by inserting it into the previous equation, we get $\beta = \pm\sqrt{\frac{y^{(k)T}s^{(k)}}{s^{(k)T}B^{(k)}s^{(k)}}}$. We now have an explicit expression for L^* and simplifying $B^{(k+1)} \equiv B^* \equiv L^* L^{*T}$ we get the BFGS approximation eq 22.

References

(1) Lew-Yee, J. F. H.; del Campo, J. M.; Piris, M. Electron Correlation in the Iron(II) Porphyrin by Natural Orbital Functional Approximations. *Journal of*

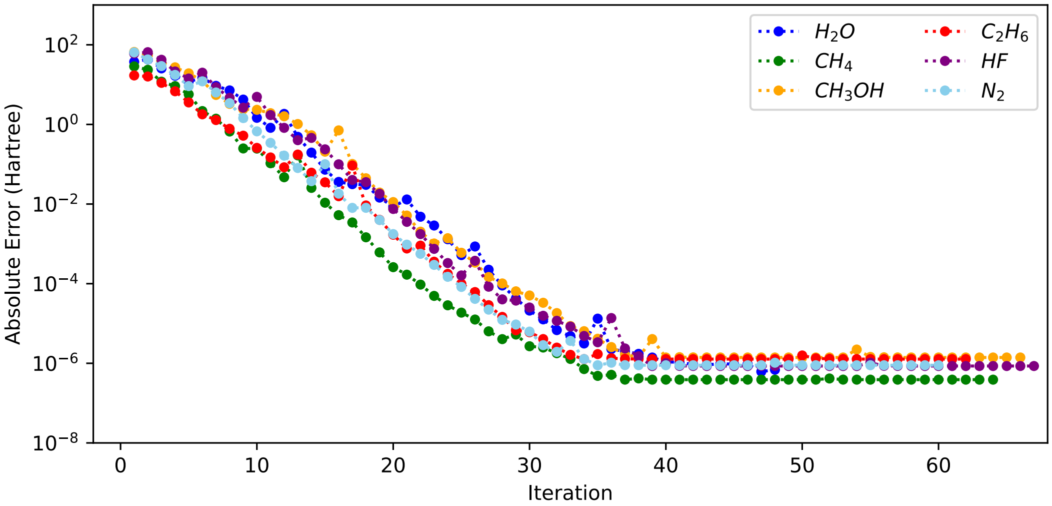

Figure 25: Convergence of the energy ($E^{(k)} - E^{(\mathrm{ref})}$ given by the Hartree–Fock functional with respect to microiteration count) using the exact Hessian.

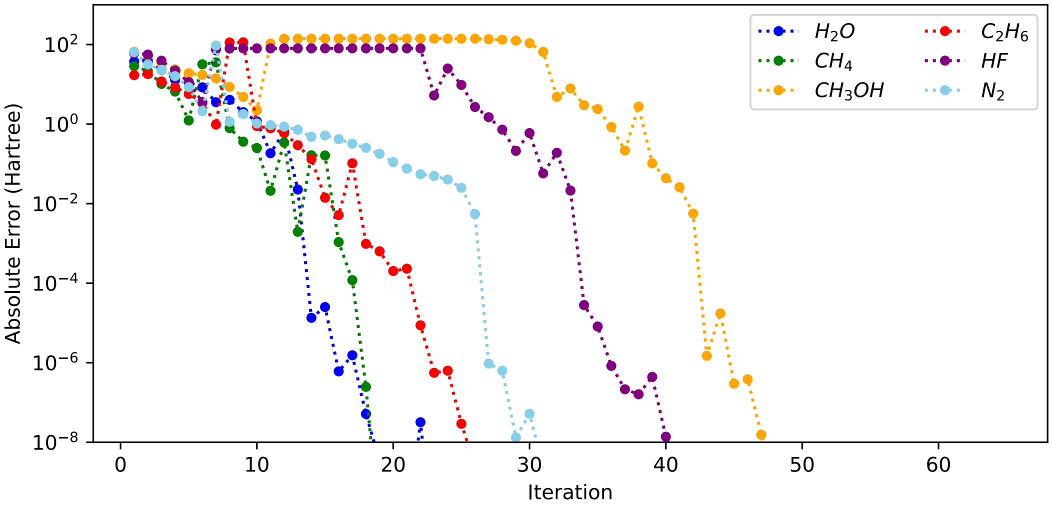

Figure 26: Convergence of the energy ($E^{(k)} - E^{(\mathrm{ref})}$ given by the Hartree–Fock functional with respect to microiteration count) using the exact Hessian. Here the boundary constraints on the ONs were handle using eq 49 instead of EBI. Note that the energy jumps for HF and C_3H_8 around 10 iterations are due to an instability in the code, which sometimes fails to impose the trace constraint $\mathrm{Tr}\{\gamma\} = N$.

Chemical Theory and Computation **2023**, *19*, 211–220, PMID: 36579972.

(2) Sharma, S.; Dewhurst, J. K.; Lathiotakis, N. N.; Gross, E. K. U. Reduced density matrix functional for many-electron systems. *Phys. Rev. B* **2008**, *78*, 201103.

(3) Lathiotakis, N. N.; Sharma, S.; Dewhurst, J. K.; Eich, F. G.; Marques, M. A. L.; Gross, E. K. U. Density-matrix-power functional: Performance for finite systems and the homogeneous electron gas. *Phys. Rev. A* **2009**, *79*, 040501.

(4) Putaja, A.; Räsänen, E. Constraints of reduced density-matrix functional theory for the two-dimensional homogeneous electron gas. *Phys. Rev. B* **2011**, *84*, 035104.

(5) Kamil, E.; Schade, R.; Pruschke, T.; Blöchl, P. E. Reduced density-matrix functionals applied to the Hubbard dimer. *Phys. Rev. B* **2016**, *93*, 085141.

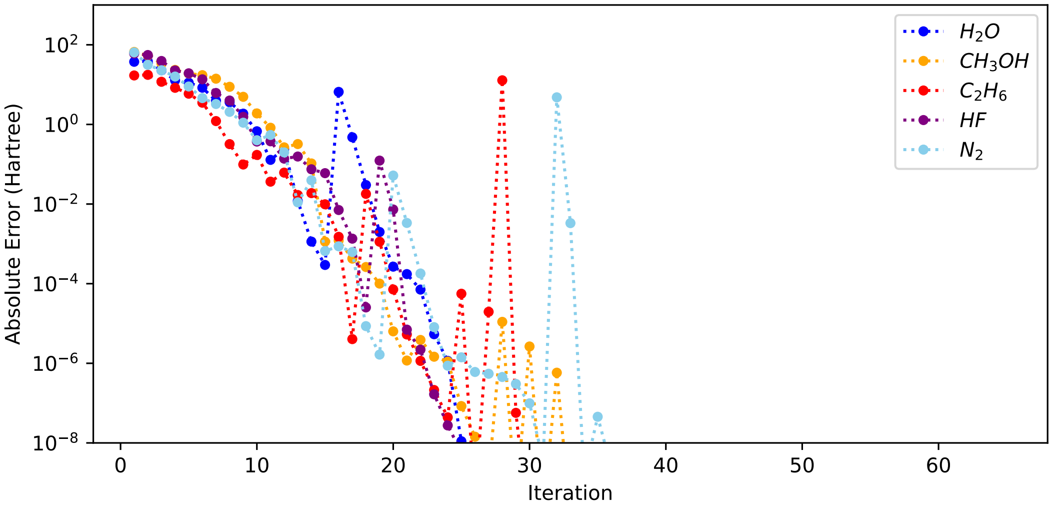

Figure 27: Convergence of the energy ($E^{(k)} - E^{(\text{ref})}$ given by the Hartree–Fock functional with respect to microiteration count) using $H_{\boldsymbol{x}}^{\text{cheap}}$ exactly and eq 27 to approximate H^{exp} with the prefactor $A(s_n, 10^{-3})$ (see text in the article). Here the boundary constraints on the ONs were handle using eq 49 instead of EBI. The result for CH_4 was not reported has is failed to impose the trace constraint $\text{Tr}\{\gamma\} = N$.

(6) Lieb, E. H. Variational Principle for Many-Fermion Systems. *Phys. Rev. Lett.* **1981**, *46*, 457–459.

(7) Veeraraghavan, S.; Mazziotti, D. A. Global solutions of Hartree-Fock theory and their consequences for strongly correlated quantum systems. *Phys. Rev. A* **2014**, *89*, 010502.

(8) Cancès, E.; Pernal, K. Projected gradient algorithms for Hartree-Fock and density matrix functional theory calculations. *The Journal of Chemical Physics* **2008**, *128*, 134108.

(9) Hauser, R. Section: Continuous Optimisation Lectrue 4: Quasi-Newton Methods. University of Oxford, Honour School of Mathematics, 2005.
